# Genome Features and Secondary Metabolites Biosynthetic Potential of the Class *Ktedonobacteria*

**DOI:** 10.3389/fmicb.2019.00893

**Published:** 2019-04-26

**Authors:** Yu Zheng, Ayana Saitou, Chiung-Mei Wang, Atsushi Toyoda, Yohei Minakuchi, Yuji Sekiguchi, Kenji Ueda, Hideaki Takano, Yasuteru Sakai, Keietsu Abe, Akira Yokota, Shuhei Yabe

**Affiliations:** ^1^Graduate School of Agricultural Science, Tohoku University, Sendai, Japan; ^2^Faculty of Agriculture, Tohoku University, Sendai, Japan; ^3^Comparative Genomics Laboratory, National Institute of Genetics, Mishima, Japan; ^4^Biomedical Research Institute, National Institute of Advanced Industrial Science and Technology, Tsukuba, Japan; ^5^Life Science Research Center, College of Bioresource Sciences, Nihon University, Fujisawa, Japan; ^6^Hazaka Plant Research Center, Kennan Eisei Kogyo Co., Ltd., Miyagi, Japan

**Keywords:** class *Ktedonobacteria*, whole genome sequencing, phylogenetic analysis, genome comparisons, NRPS, PKS, antimicrobial screening

## Abstract

The prevalence of antibiotic resistance and the decrease in novel antibiotic discovery in recent years necessitates the identification of potentially novel microbial resources to produce natural products. *Ktedonobacteria*, a class of deeply branched bacterial lineage in the ancient phylum *Chloroflexi*, are ubiquitous in terrestrial environments and characterized by their large genome size and complex life cycle. These characteristics indicate *Ktedonobacteria* as a potential active producer of bioactive compounds. In this study, we observed the existence of a putative “megaplasmid,” multiple copies of ribosomal RNA operons, and high ratio of hypothetical proteins with unknown functions in the class *Ktedonobacteria*. Furthermore, a total of 104 antiSMASH-predicted putative biosynthetic gene clusters (BGCs) for secondary metabolites with high novelty and diversity were identified in nine *Ktedonobacteria* genomes. Our investigation of domain composition and organization of the non-ribosomal peptide synthetase and polyketide synthase BGCs further supports the concept that class *Ktedonobacteria* may produce compounds structurally different from known natural products. Furthermore, screening of bioactive compounds from representative *Ktedonobacteria* strains resulted in the identification of broad antimicrobial activities against both Gram-positive and Gram-negative tested bacterial strains. Based on these findings, we propose the ancient, ubiquitous, and spore-forming *Ktedonobacteria* as a versatile and promising microbial resource for natural product discovery.

## Introduction

The discovery and clinical application of antimicrobial drugs have greatly improved our well-being with regard to microbial infection over the past several decades (Demain and Sanchez, 2009). However, misuse and overuse of antibiotics has led to the accelerating development of antibiotic resistance in pathogens, which has become a major threat to modern health care and the natural environment (Pal et al., 2016; Jiang et al., 2017). Traditionally, *Actinobacteria*, particularly the genus *Streptomyces*, has served as the major producer of antimicrobial natural products, contributing 62% of the microbe-derived antibiotics from the 1950s to 1970s (Bérdy, 2012). In contrast, the ratio has decreased to 25% in the 2000s to 2010s, with the silencing of gene clusters encoding biosynthetic enzymes required for natural products under laboratory conditions further hampering the discovery of novel antibiotics via the traditional bacterial fermentation method (Rutledge and Challis, 2015). Given that, isolation and screening of rare actinomycetes and marine-derived actinomycetes have become popular. Furthermore, the application of *in silico* biosynthetic predictions from genome mining data and novel developed heterologous expression technologies confirm the phylum *Actinobacteria* as a continuing source of novel antibiotics (Bérdy, 2012; Genilloud, 2017). Nevertheless, it is of high risk to focus the discovery of novel natural products on only a single phylum as this source could become exhausted. Thus, the exploration of new microbial resources comparable to actinomycetes is extremely important.

Numerous bioactive natural products are derived from bacterial secondary metabolites, with the enzymes required for biosynthesis of these natural products being usually encoded on certain bacterial genome loci termed biosynthetic gene clusters (BGCs). Traditionally, BGCs include non-ribosomal peptide synthase (NRPS), polyketide synthase (PKS), and ribosomally synthesized and post-translationally modified peptide (RiPP) family clusters. The BGCs of NRPS and PKS encode modular enzymes that comprise functional synthesis blocks known as modules. Therefore, NRPSs and PKSs constitute a large class of structurally diverse and biomedically useful bacterial secondary metabolites and have accordingly gained considerable attention in recent years (Doroghazi and Metcalf, 2013; Bitok et al., 2017; Vila-Farres et al., 2017). Typical NRPSs contain three essential domains: an AMP-binding (A) domain responsible for the selection and activation of monomers, a peptidyl carrier protein (PCP) domain, also termed the thiolation (T) domain, required for attachment and transfer of the adenylated monomer, and a condensation (C) domain for catalyzing amide bond formation and chain elongation (Challis and Naismith, 2004). PKS pathways share some similarities with fatty acid synthase (Dutta et al., 2014) and can be further divided into three types: type I PKSs that comprise large successive modular enzymes containing multi-functional domains; type II PKSs constituting single or bifunctional enzymes; and type III PKSs that utilize co-enzyme A-tethered substrates rather than the acyl carrier protein (ACP) domain to attach and transfer monomers such as type I and type II PKSs (Shen, 2003). Similar to NRPSs, a typical type I PKS also comprises three essential domains: an acyltransferase (AT) domain, which transfers a malonyl group from malonyl-CoA onto the 40-phosphopantetheine prosthetic group of an ACP domain, and a ketosynthase (KS) domain responsible for elongation of the polyketide chain (Masschelein et al., 2017). Moreover, similarities shared by NRPSs and PKSs in their biosynthesis pathway lead to hybrid non-ribosomal peptide-polyketides, which further enlarge the diversity of bacterial natural products (Miller et al., 2002; Liu et al., 2004; Shou et al., 2016). RiPPs also represent another type of antimicrobial peptide and are being considered as promising alternative antimicrobial agents owing to their widespread bactericidal sensitivity (Lázár et al., 2018). RiPPs are divided into different subfamilies according to their biosynthetic machinery and structural features, such as lantipeptides, lasso peptides, thiopeptides, and bacteriocins (Ortega and van der Donk, 2016). However, despite the differences in classification, almost all RiPPs contain an N-terminal leader peptide and a C-terminal core peptide (Ortega and van der Donk, 2016). Additionally, unlike NRPSs and PKSs, RiPPs always follow a simple biosynthesis pathway wherein the leader peptide guides the post-translational modification of the precursor peptide and is removed by one or more peptidases in the final step of core peptide maturation (Letzel et al., 2014; Ortega and van der Donk, 2016).

The class *Ktedonobacteria* was first reported in 2006 and currently contains two orders, three families, four genera, and seven formally proposed species including both mesophilic (Cavaletti et al., 2006; Yabe et al., 2017b) and thermophilic strains (Yabe et al., 2010, 2011). Owing to the morphological similarities, *Ktedonobacter racemifer* SOSP1-21^T^, the first proposed *Ktedonobacteria* species, was initially mistakenly identified as an actinomycete (Cavaletti et al., 2006). However, phylogenetic analysis based on 16S rRNA gene sequences placed the class *Ktedonobacteria* in the phylum *Chloroflexi* (Cavaletti et al., 2006; Yabe et al., 2010). Similar to actinomycetes, isolates and environmental DNA belonging to this class were detected to be ubiquitous in various terrestrial environments from common soil (Cavaletti et al., 2006; Yabe et al., 2017b), to extreme environments, such as an acid vapor-formed spring (Jiang et al., 2016), dark oligotrophic volcanic ice cave ecosystems of Mt. Erebus in Antarctica (Tebo et al., 2015), naturally occurring CO_2_ gas vents (de Miera et al., 2014b), recently deglaciated high-altitude soils of the Himalaya (Stres et al., 2013), and a lava cave in a volcanic trench (Northup et al., 2011). In addition, comparable to fungi, most *Streptomyces* species inhabit the soil environment and live as saprophytic bacteria (Flärdh and Buttner, 2009). In contrast, unlike actinomycetes, the class *Ktedonobacteria* appears to preferentially predominate in oligotrophic and extreme environments, suggesting these bacteria may possess different metabolic pathways from actinomycetes.

**FIGURE 1:**
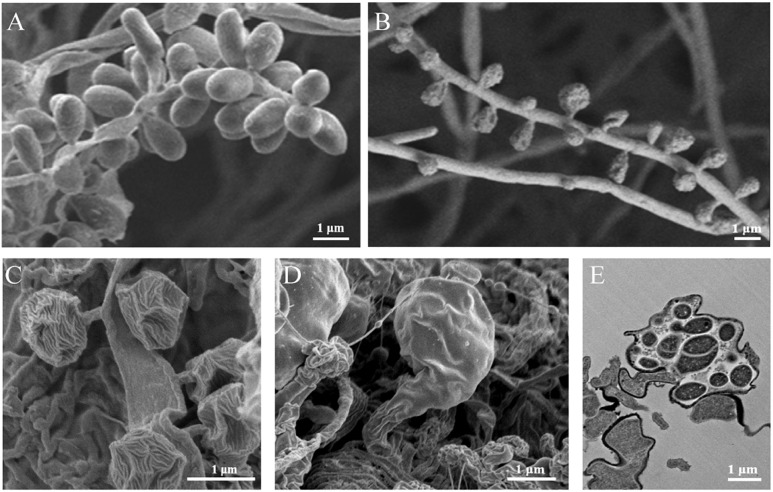
Morphologies of the isolated *Ktedonobacteria* strains. Scanning electron micrographs of strain *Thermosporothrix hazakensis* SK20-1^T^ **(A)**, *Thermogemmatispora onikobensis* ONI-1^T^ **(B)**, *Ktedonobacter racemifer* SOSP1-21^T^ **(C)**, and *Dictyobacter aurantiacus* S27^T^ **(D)**, indicating the exospores **(A–C)** or sporangia **(D,E)** formed by these strains. **(E)** Transmission electron micrographs of the thin-sectioned sporangium formed by strain *Dictyobacter aurantiacus* S27^T^. Bars: 1 μm.

Notably, as shown in Figure 1, some isolated *Ktedonobacteria* strains exhibit complex morphologies. The genus *Thermosporothrix* forms substrate mycelia and subsequently produces grape-like smooth exospores by budding on the aerial mycelia (Yabe et al., 2010), whereas the surfaces of exospores formed by the genus *Thermogemmatispora* are wrinkled (Yabe et al., 2011). In contrast, strain *K. racemifer* SOSP1-21^T^ forms spherical spores with a dried plum shape on aerial mycelia (Cavaletti et al., 2006), whereas *Dictyobacter aurantiacus* S27^T^ produces globose or subglobose terminal sporangia from the vegetative mycelia via short stalk cells (Yabe et al., 2019). Spore formation represents one of the numerous methods developed by bacteria to survive in conditions of nutrient depletion or harsh environment (Errington, 2003; Flärdh and Buttner, 2009). It has been suggested that bacteria with complex morphological differentiation may possess an active secondary metabolism (Flärdh and Buttner, 2009; Manivasagan et al., 2014). Previously, metabolites of new acyloins and thiazoles have been identified in the fermentation broth of *Thermosporothrix hazakensis* SK20-1^T^ (Park et al., 2014, 2015), whereas the extremophilic *Thermogemmatispora* strain T81 produced a four (methyl) lanthionine-bridged new lanthipeptide that showed antimicrobial activity against the human pathogen *Staphylococcus aureus* (Xu et al., 2018). Furthermore, the released genome draft data revealed that the genome sizes of strain *Thermogemmatispora onikobensis* ONI-1^T^ (Komaki et al., 2016) and *Thermogemmatispora carboxidivorans* PM5^T^ were 5.56 and 5.61 Mb, respectively, whereas strain *K. racemifer* SOSP1-21^T^ supported a large genome of 13.66 Mb (Chang et al., 2011), which is the second largest bacterial genome identified to date following that of *Sorangium cellulosum* So0157-2 (14.78 Mb) (Han et al., 2013).

Together, these described characteristics encouraged us to consider the class *Ktedonobacteria* as a Gram-positive, novel microbial resource for bioactive natural compounds discovery. However, the phylogenetic position and genome features of this class remained unclear. Moreover, although the large genome size of the *Ktedonobacteria* species suggest that they might encode multiple secondary metabolism gene clusters (Omura et al., 2001; Li and Walsh, 2010), few descriptions of these gene clusters or their novel natural product biosynthetic potential have been published. In this study, we therefore performed genome sequencing for another six *Ktedonobacteria* strains. The total nine available *Ktedonobacteria* genomes were then subjected to comprehensive phylogenetic, genomic, and secondary metabolic analysis, revealing the unique genome features and diversity of the predicted BGCs. Antimicrobial activity performance of the nine studied *Ktedonobacteria* strains against a variety of bacterial strains was also assessed.

## Materials and Methods

### Strain Cultural Conditions

Origins of the strains utilized in this study are described in Section “Strain Information and Whole Genome Sequencing.” *Ts. hazakensis* SK20-1^T^ and *Ts. hazakensis* COM3 were maintained in pure culture at 50°C on ISP3 agar plates (Shirling and Gottlieb, 1966), whereas the other seven strains were maintained on 1/10 R2A gellan gum (Kanto Chemical Co., Inc., Tokyo, Japan) plates (Yabe et al., 2017b). The optimal growth temperature for *K. racemifer* SOSP1-21^T^, *D. aurantiacus* S27^T^, *Ktedonobacterales* bacterium Uno3, and *Ktedonobacterales* bacterium Uno16 was 30°C, whereas *Tg. onikobensis* ONI-1^T^, *Thermogemmatispora* sp. A3-2, and *Tg. carboxidivorans* PM5^T^ were cultured at 60°C. For genomic DNA extraction, strains *Ts. hazakensis* SK20-1^T^ and *Ts. hazakensis* COM3 were cultured at 50°C in ISP1 liquid medium (Shirling and Gottlieb, 1966). Strain *Thermogemmatispora* sp. A3-2 was cultured at 65°C, whereas strains *D. aurantiacus* S27^T^, *Ktedonobacterales* bacterium Uno3, and *Ktedonobacterales* bacterium Uno16 were cultured at 30°C in 1/10 R2A liquid medium (Yabe et al., 2017b).

The candidate testing microorganisms used for antimicrobial assays included *Bacillus subtilis* NBRC 3134, *Pseudomonas aeruginosa* NBRC 13275, *Escherichia coli* NBRC 3972, *Salmonella enterica* NBRC 100797, *Micrococcus luteus* NBRC 13867, *Geobacillus stearothermophilus* NBRC 13737, *Staphylococcus aureus* NBRC 13276, *S. aureus* NTCT8325 (MSSA) and *S. aureus* Mu50 (VISA) (Kuroda et al., 2001) were provided by Juntendo University. All strains were maintained in nutrient agar plates.

### Genomic DNA Extraction and Whole Genome Sequencing

To extract genomic DNA from the *Ktedonobacteria* strains, a modified method following the protocol of the Puregene Yeast/Bact. Kit B (Qiagen, Hilden, Germany) was used. The bacterial cells were collected by centrifugation of the culture broth and an appropriate volume of purified achromopeptidase (Fujifilm Wako Pure Chemical Corporation, Osaka, Japan) and lysozyme (MP Biomedicals, LLC, Illkirch, France) were added to the tubes to break down the cell walls. Also, 10 μL of 20 mg/mL proteinase K was added and incubated at 55°C for 1 h, then the tubes were incubated at 37°C overnight gently in a shaker to digest the residual proteins. Finally, the dried DNA pellets were dissolved in 100 μL DNA hydration solution for genome sequencing or stored at -20°C for later use.

Genome sequencing of strain *Ts. hazakensis* SK20-1^T^ was conducted by TaKaRa Bio Inc., Japan on a PacBio *RS* II platform with long sequencing and assembled *de novo* using SMRT Analysis v2.2.0^1^. The genome sequences of strain *Ts. hazakensis* COM3 and *Thermogemmatispora* sp. A3-2 were obtained from paired-end sequencing with HiSeq 2500 and long sequencing with PacBio *RS* II/Sequel. The genome sequencing was conducted by the National Institute of Genetics in Mishima, Shizuoka, Japan. The reads were assembled *de novo* using HGAP3/4^2^, and gaps between contigs were closed with their fosmid clones. Genome data of strains *K. racemifer* SOSP1-21^T^, *D. aurantiacus* S27^T^, *Ktedonobacterales* bacterium Uno3, *Ktedonobacterales* bacterium Uno16, *Tg. onikobensis* ONI-1^T^, and *Tg. carboxidivorans* PM5^T^ were recovered from NCBI and DDBJ databases for analysis. Additionally, the qualities (genome completeness and contamination) of the nine studied *Ktedonobacteria* genomes were estimated by CheckM, a set of tools using collocated sets of genes that are ubiquitous and single-copy within a phylogenetic lineage (Parks et al., 2015). As the class *Ktedonobacteria* is a relatively novel taxonomy of bacteria, both the lineage-specific workflow, taxonomic-specific workflow, and 83 single copy custom marker genes (Soo et al., 2014) were used in the CheckM estimation.

### Phylogenetic Analysis Based on 16S rRNA Genes and Single-Copy Marker Genes

For phylogenetic analysis, *Chloroflexi*-related 16S rRNA gene sequences were obtained from the All-species Living Tree Project (LTP, release s132) (Yarza et al., 2010). The 16S rRNA gene sequence of *T. hazakensis* COM3 was extracted from its genome sequence (determined in this study) using CommunityM^3^ and aligned via the ARB software package (Ludwig et al., 2004) using the LTP database as a reference; the alignment was manually corrected using the ARB EDIT tool of the ARB package. Sequences (>1,300 nt) representing various family-level taxa in the phylum were selected in ARB and their alignments exported with lane mask filtering. Neighbor joining trees based on LogDet distance were computed using PAUP^∗^4 (Swofford, 2003) with 100 bootstrap resamplings. Maximum likelihood trees were built using RAxML v8.2 (GTR and Gamma models + I) with rapid 100 times bootstrapping (Stamatakis, 2006). The trees were visualized and inspected in ARB.

Phylogenetic relationships among members of the phylum *Chloroflexi*, including members of the class *Ktedonobacteria*, were assessed using the genome sequences determined in the present study along with other finished and draft genomes representing the major families in the phylum as described in Sun et al. (2016). Briefly, 38 (Darling et al., 2014) or 83 (Soo et al., 2014) single-copy marker gene products were extracted using hidden Markov model searches as described previously (Sekiguchi et al., 2015). Tree topologies were tested for robustness using the maximum likelihood methods from FastTree v2 (with default parameters, JTT model, CAT approximation) (Price et al., 2010) and RAxML v8.2 (JTT and Gamma models with rapid 100 times bootstrapping).

### Genome Annotation and Comparisons

The nine *Ktedonobacteria* genomes and related genomes were primarily annotated using DFAST (DDBJ Fast Annotation and Submission Tool) (Tanizawa et al., 2018) to study their general features. The DFAST-annotated 16S rRNA gene sequences were extracted and blasted using NCBI Nucleotide-Nucleotide Blast^4^ program to compare their similarities. Meanwhile, the Kyoto Encyclopedia of Genes and Genomes (KEGG) PATHWAY database was used for functional characterization of the nine genomes (Moriya et al., 2007).

CGView Server (Grant and Stothard, 2008) and the Geneious prime 2019.0.4 software were used to generate the genome plot. To compare the genomic rearrangement among *Ktedonobacteria* species, the contigs were concatenated using the Geneious software. Then the concatenated genomic sequences were aligned with the ProgressiveMauve algorithm and visualized using the MAUVE plugin within Geneious suite (Darling et al., 2004). BPGA (Bacterial Pan Genome Analysis tool), an ultra-fast pan-genome analysis pipeline (Chaudhari et al., 2016), was used to compare genomes between different strains in the class *Ktedonobacteria* and with other classes.

### Metabolites Analysis and Identification of Putative BGCs

The nine *Ktedonobacteria* genomes were submitted to antiSMASH version 4.2.0 (antibiotics and Secondary Metabolites Analysis Shell) (Blin et al., 2017) and the integrated ClusterFinder algorithm, an hidden Markov model based probabilistic algorithm to detect BGC-like regions in genomes of unknown types (Cimermancic et al., 2014), to identify both the characterized and unknown secondary metabolite biosynthesis gene clusters in the nine *Ktedonobacteria* strains. Additionally, both the KnownClusterBlast and ClusterBlast modules were selected to identify similar clusters in sequenced genomes by genome comparisons. Domain functions and genetic similarities with known BGCs in these gene clusters were further predicted and annotated using Protein-Protein BLAST and Pfam analyses (Finn et al., 2016). The AMP-binding domain amino acid substrate specificities were predicted with antiSMASH referencing three prediction algorithms, NRPSPredictor2 (Röttig et al., 2011), Stachelhaus code (Stachelhaus et al., 1999), and SANDPUMA ensemble (Chevrette et al., 2017). Only when two or all three algorithms provided consistent substrate specificity predictions was a consensus prediction called. Otherwise, the prediction results were assigned an “X” to represent an unknown amino acid residue, or all of the dubious amino acid residues were listed (Chu et al., 2016).

### Phylogenetic Analysis of the PKS KS Domain, NRPS C Domain, and Lantipeptide Modification Genes

All of the PKS KS domains and NRPS C domains identified in putative BGCs from *Ktedonobacteria* genomes were extracted and submitted to NCBI Protein-Protein Blast (see text footnote 4). The top three reference sequences, excluding the *Ktedonobacteria* sequences, were selected and preserved in the FASTA file. Sequences of the *Ktedonobacteria* PKS KS domains, NRPS C domains, and the corresponding reference sequences were then submitted to Natural Product Domain Seeker (NaPDoS) (Ziemert et al., 2012) for functional classification. For phylogenetic tree construction of the KS and C domains, the sequences were trimmed into equal length by MEGA7.0^5^, and the tree was constructed following the method described in Section “Phylogenetic Analysis Based on 16S rRNA Genes and Single-Copy Marker Genes” for 16S rRNA phylogenetic analysis. Additionally, the phylogenetic trees were displayed and annotated by iTOL v3, an online tool for the display, annotation, and management of phylogenetic trees (Letunic and Bork, 2016).

The lantipeptide modification genes and precursor peptides were identified via a comprehensive analysis of antiSMASH 4.2.0 and BAGEL 3.0, a web-based comprehensive mining suite to identify and characterize RiPPs in microbial genomes (van Heel et al., 2013). Reference sequences of modification genes were obtained from the NCBI database with the same method as for KS domains and C domains. The phylogenetic trees were also constructed, displayed, and annotated following the aforementioned methods. For the analysis of precursor peptides, the extracted sequences were aligned with MEGA7.0 and the figures indicating cleavage sites were visualized using WebLogo 3 (Crooks et al., 2004).

### Antimicrobial Screening of the Class *Ktedonobacteria*

Strains *Ts. hazakensis* COM3 and *Thermogemmatispora* sp. A3-2 were selected as representatives of the genus *Thermosporothrix* and *Thermogemmatispora*, respectively. Strain *Ts. hazakensis* COM3 was cultured in basic medium (peptone 2 g/L, yeast extract 1 g/L, MgSO_4_ 1 g/L, NaCl 1 g/L, and pH 7.0) at 50°C for 7 days, whereas strain *Thermogemmatispora* bacterium A3-2 was cultured in R2A medium at 60°C for 7 days. Strains *K. racemifer* SOSP1-21^T^, *Dictyobacter aurantiacus* S27^T^, *Ktedonobacterales* Uno3, and *Ktedonobacterales* Uno16 were cultured in Bennett’s medium (yeast extract 1 g/L, beef extract 1 g/L, N-Z amine type A 2 g/L, maltose 10 g/L, and pH 7.0) at 30°C for 14 days. Additionally, 2% Diaion^®^ HP-20 polyaromatic adsorbent resin was added to each medium. Following 2 L culture, the cell pellets and HP-20 polyaromatic adsorbent resin were collected and extracted with the appropriate volume of acetone. Extractions from the above six strains were dried via evaporator and then redissolved in 5 mL of 80% methanol (MeOH) for antimicrobial activity screening versus a series of candidate testing microorganisms.

The antimicrobial activity screening of the class *Ktedonobacteria* was performed using the standard paper disc method. Paper discs (Toyobo Co., Ltd., Osaka, Japan; 6 mm) were saturated with 10 μL bacterial extracts from the above *Ktedonobacteria* strains and dried at room temperature. Then, the paper discs were placed on the surface of agar plates inoculated with each testing candidate microorganism and incubated for 24 h at the optimal temperature. Results of antimicrobial activity were determined by measuring diameters of zones of inhibition formed around each paper disc by the bacterial extracts. All antimicrobial tests were performed in triplicate.

## Results

### Strain Information and Whole Genome Sequencing

As summarized in Table 1, the nine described *Ktedonobacteria* isolates were all terrestrially derived, including five thermophilic strains and four mesophilic strains. *Ts. hazakensis* COM3 was isolated from the same compost with *Ts. hazakensis* SK20-1^T^ (Yabe et al., 2010) and was identified as a homologous strain to *Ts. hazakensis* SK20-1^T^ by 16S rRNA gene sequence. However, *Ts. hazakensis* COM3 was distinguished from *Ts. hazakensis* SK20-1^T^ through the formation of red pigment in culture medium (unpublished), and thus was also studied for genome analysis in this study. Strain *Thermogemmatispora* sp. A3-2 was isolated from the same geothermal soils as *Tg. onikobensis* ONI-1^T^ and was identified as a novel species belonging to the genus *Thermogemmatispora*. *Thermogemmatispora* sp. A3-2 is preserved in our laboratory and in the Biological Resource Center, NITE (NBRC) and China General Microbiological Culture Collection (CGMCC). Moreover, *Thermogemmatispora* sp. A3-2 grew at 40–78°C, the highest growth temperature in the class *Ktedonobacteria* identified to date. *Ktedonobacterales* bacterium Uno3 and *Ktedonobacterales* bacterium Uno16 were isolated from soil-like microbe masses termed “Tengu-no-mugimeshi” at a volcanic region located in Gunma, Japan (Okada, 1937). Optimal growth temperature of the two isolates was 30°C.

**Table 1:** Strain information of the nine studied *Ktedonobacteria* strains.

Order	Strain	Isolation source	Growth temperature (optimal)	Morphology	References
*Ktedonobacterales*	*Dictyobacter aurantiacus* S27^T^	Waterlogged rice paddy field	20∼37°C (25∼30°C)	Sporangia on aerial mycelia	Yabe et al., 2017b
	*Ktedonobacterales* bacterium Uno16	Soil-like microbe masses	11∼37°C (20∼30°C)	Exospores on aerial mycelia	Wang et al., 2019
	*Ktedonobacterales* bacterium Uno3	Soil-like microbe masses	20∼37°C (20∼30°C)	Exospores on aerial mycelia	Wang et al., 2019
	*Ktedonobacter racemifer* SOSP1-21^T^	Soil	17∼40°C (28∼33°C)	Exospores on aerial mycelia	Cavaletti et al., 2006
	*Thermosporothrix hazakensis* SK20-1^T^	Ripe compost for livestock treatment	31∼58°C (50°C)	Exospores on aerial mycelia	Yabe et al., 2010
	*Thermosporothrix hazakensis* COM3	Ripe compost for livestock treatment		Exospores on aerial mycelia	This study
*Thermogemmatisporales*	*Thermogemmatispora onikobensis* ONI-1^T^	Geothermal soils	50∼74°C (60∼65°C)	Exospores on aerial mycelia	Yabe et al., 2011
	*Thermogemmatispora carboxidivorans* PM5^T^	Geothermally heated biofilm	40∼65°C (55°C)	Exospores on aerial mycelia	King and King, 2014
	*Thermogemmatispora* sp. A3-2	Geothermal soils	40∼78°C (60∼65°C)	Exospores on aerial mycelia	This study


Prior to this study, the draft genome sequences of *K. racemifer* SOSP1-21^T^ (Chang et al., 2011), *Tg. onikobensis* ONI-1^T^ (Komaki et al., 2016), and *Tg. carboxidivorans* PM5^T^ have been publically released. In addition, genome sequencing of *D. aurantiacus* S27^T^ (GenBank accession: BIFQ01000001 and BIFQ01000002), *Ktedonobacterales* bacterium Uno3 (GenBank accession: BIFR01000001 and BIFR01000002), and *Ktedonobacterales* bacterium Uno16 (GenBank accession: BIFT01000001, BIFT01000002, BIFT01000003, and BIFT01000004) was performed by our group in another study (Wang et al., 2019) and the genome data were deposited in IMG or DDBJ/EMBL/GenBank databases. In the present study, we conducted genome sequencing for three strains including *Ts. hazakensis* SK20-1^T^ (GenBank accession: BIFX01000001, BIFX01000002, and BIFX01000003), *Ts. hazakensis* COM3 (GenBank accession: AP019376), and *Thermogemmatispora* sp. A3-2 (GenBank accession: AP019377). Sequencing platforms, sequence coverage, assembly methods, and accession numbers of the nine *Ktedonobacteria* genomes are summarized in Supplementary Table S1.

**FIGURE 2:**
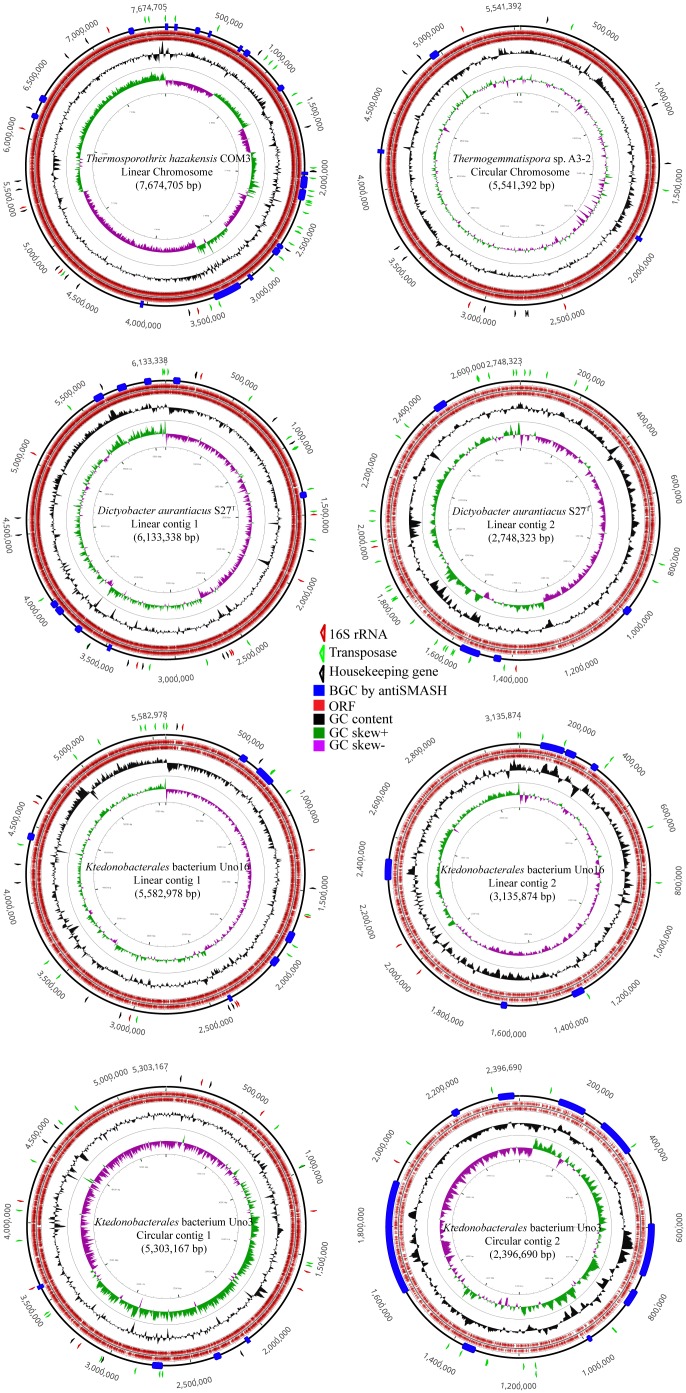
Genome plot of strains of *Thermosporothrix hazakensis* COM3, *Thermogemmatispora* sp. A3-2, *Dictyobacter aurantiacus* S27^T^, *Ktedonobacterales* bacterium Uno16, and *Ktedonobacterales* bacterium Uno3. The out layer triangle markers indicate the 16S rRNA gene in red, transfase in green, and part of the housekeeping genes (gmk, rpoD, recA, glyA, dnaB, gyrA, gyrB, secA, dnaK, ffh) in black. The inner layer colored boxes indicate the antiSMASH-identified BGCs in blue, ORFs in red, GC content in black, GC skew+ in green, and GC skew– in purple.

As shown in Figure 2, draft genome of *Ktedonobacterales* bacterium Uno3 possesses two circular contigs (5.30 Mb and 2.40 Mb, respectively) whereas the complete genome of *Thermogemmatispora* sp. A3-2 comprises a single circular chromosome. *Ts. hazakensis* COM3 possesses one putative linear chromosome, whereas *D. aurantiacus* S27^T^ contains two linear contigs (6.13 Mb and 2.75 Mb, respectively). *Ktedonobacterales* bacterium Uno16 comprises two linear contigs (5.58 Mb and 3.14 Mb) and two circular plasmids of 199,150 and 43,380 bp in size, respectively. Initially, the second circular contig of *Ktedonobacterales* bacterium Uno3 (2.40 Mb) and the second linear contigs of strains *D. aurantiacus* S27^T^ (2.75 Mb) and *Ktedonobacterales* bacterium Uno16 (3.14 Mb) were thought to be part of the chromosomes because they were quite large in size compared with normal bacterial plasmids. However, the above “chromosomes” were determined to be incomplete due to the absence of most bacterial house-keeping genes in the subsequent CheckM assessment (Supplementary Table S2), suggesting that they may be “megaplasmids” (Thomas and Summers, 2008; Wu et al., 2009).

### General Genome Features

As given in Table 2 and Supplementary Table S1, genome sizes of the nine *Ktedonobacteria* strains ranged from 5.54 to 13.66 Mb, which were larger than the other classes in the phylum *Chloroflexi*. The average genome size of the genus *Thermosporothrix* and *Thermogemmatispora* was 7.48 and 5.57 Mb, respectively. Excluding strain *K. racemifer* SOSP1-21^T^, the average genome size of the three mesophilic strains including strains *D. aurantiacus* S27^T^, *Ktedonobacterales* bacterium Uno3, and *Ktedonobacterales* bacterium Uno16 was 8.51 Mb. However, the nine *Ktedonobacteria* strains exhibited a much lower GC content (an average of 55.1%) than that of actinomycete strains, especially the genus *Streptomycetes* (Chater and Chandra, 2006). Additionally, the class *Ktedonobacteria* encoded multiple copies of ribosomal RNA operons (8∼28 copies per genome) and 16S rRNA genes (3∼9 copies per genome) on their genomes. The data of *Tg. onikobensis* ONI-1^T^ was not counted due to the poor genome quality. In addition, a maximum of 1.49% 16S rRNA gene variation was observed in strain *D. aurantiacus* S27^T^ (Supplementary Table S3). Moreover, 2364 (strain *Tg. onikobensis* ONI-1^T^) to 8227 (strain *K. racemifer* SOSP1-21^T^) of the detected coding sequences (CDSs) were termed as hypothetical proteins with unknown functions by DFAST annotation (Tanizawa et al., 2018), representing an average of 57.0% of the total CDSs in the nine *Ktedonobacteria* strains.

**Table 2:** General genome features of the nine studied *Ktedonobacteria* strains.

Strain	Genome size (Mb)	Sequences	Completeness (%)	G + C (mol%)	CDSs	rRNA	tRNA	Hypothetical protein	Transposase
*D. aurantiacus* S27^T^	8.88	Linear contig 1 (6.13 Mb)	98.3	54.4	5137	23	63	2873	17
		Linear contig 2 (2.75 Mb)	3.3	53.0	2364	4	4	1345	36
*Ktedonobacterales* bacterium Uno16	8.96	Linear contig 1 (5.58 Mb)	99.3	49.8	4665	21	61	2665	17
		Linear contig 2 (3.14 Mb)	3.6	49.2	2663	7	2	1572	8
		Circular plasmid 1 (0.20 Mb)	–	53.7	2030	0	0	194	0
		Circular plasmid 2 (0.04 Mb)	–	52.1	48	0	1	46	0
*Ktedonobacterales* bacterium Uno3	7.70	Circular contig 1 (5.30 Mb)	98.7	49.5	4397	26	64	2568	15
		Circular contig 2 (2.40 Mb)	3.7	49.1	1836	2	0	1159	14
*K. racemifer* SOSP1-21^T^	13.66	10 contigs	100	53.8	12680	23	64	8227	424
*Ts. hazakensis* SK20-1^T^	7.40	3 contigs	98.3	53.1	6355	15	62	3599	27
*Ts. hazakensis* COM3	7.67	Linear chromosome	98.3	53.2	6569	15	63	3627	30
*Tg. onikobensis* ONI-1^T^	5.56	112 contigs	99.0	61.1	4331	6	47	2364	2
*Tg. carboxidivorans* PM5^T^	5.61	1 contig	99.0	60.9	4404	8	49	2338	2
*Thermogemmatispora* sp. A3-2	5.54	Circular chromosome	99.1	60.4	4299	8	49	2298	2


To investigate the absence/presence of genes responsible for autotrophic metabolites, the nine studied *Ktedonobacteria* genomes were submitted to the KEGG PATHWAY database for functional characterization. Additionally, the presence of a type I carbon monoxide dehydrogenases (*cox*) gene in the genome of *Tg. carboxidivorans* PM5^T^ and *Thermogemmatispora* sp. T81 was also reported and functioned in the oxidation of carbon monoxide (CO) to carbon dioxide (CO_2_) (King and King, 2014; Islam et al., 2019). Herein, we aligned amino acids sequences of the *cox* gene with the annotated genes and their homologs in the nine studied genomes, using the NCBI Protein-Protein Blast program. As given in Supplementary Table S4A, all nine *Ktedonobacteria* strains possessed multiple copies of *cox* or their homologs in their genomes. Moreover, pyruvate synthase and α-ketoglutarate synthase, key enzymes in the reductive citric acid cycle (reductive TCA cycle) (Buchanan and Arnon, 1990), were present in the nine *Ktedonobacteria* strains. However, adenosine triphosphate (ATP) citrate lyase, a key enzyme responsible for the cleaving of citrate, was absent in the *Ktedonobacteria* genomes (Supplementary Table S4B). Additionally, comparisons between contig1 and contig2 of strains *D. aurantiacus* S27^T^ and *Ktedonobacterales* bacterium Uno16 are also given in Table 3.

**Table 3:** Comparisons of contig1 and contig2 in strains *D. aurantiacus* S27^T^, *Ktedonobacterales* bacterium Uno16, and *Ktedonobacterales* bacterium Uno3.

Genes	*D. aurantiacus* S27^T^		*Ktedonobacterales* bacterium Uno16		*Ktedonobacterales* bacterium Uno3	
			
	contig1	contig2	contig1	contig2	contig1	contig2
**Essential genes for growth**						
Housekeeping genes^∗1^	104	11	104	3	104	4
Translation^∗2^	77	5	77	3	74	3
Replication and repair^∗2^	66	8	65	9	67	8
Citrate cycle (TCA cycle)^∗2^	23	1	21	1	20	3
Oxidative phosphorylation^∗2^	42	4	44	8	41	3
**Adaptation to environment**						
Transporter related genes^∗3^	223	178	213	185	179	109
Cytochrome P450 related genes^∗3^	15	17	10	18	6	13
Regulator genes^∗3^	193	128	175	159	120	82


*^∗*1*^The result of CheckM using dataset of domain bacteria. ^∗*2*^The result of KEGG pathway functional characterization. ^∗*3*^The result of DFAST annotation.*

### Phylogenetic Analysis and GenomeComparisons

**FIGURE 3:**
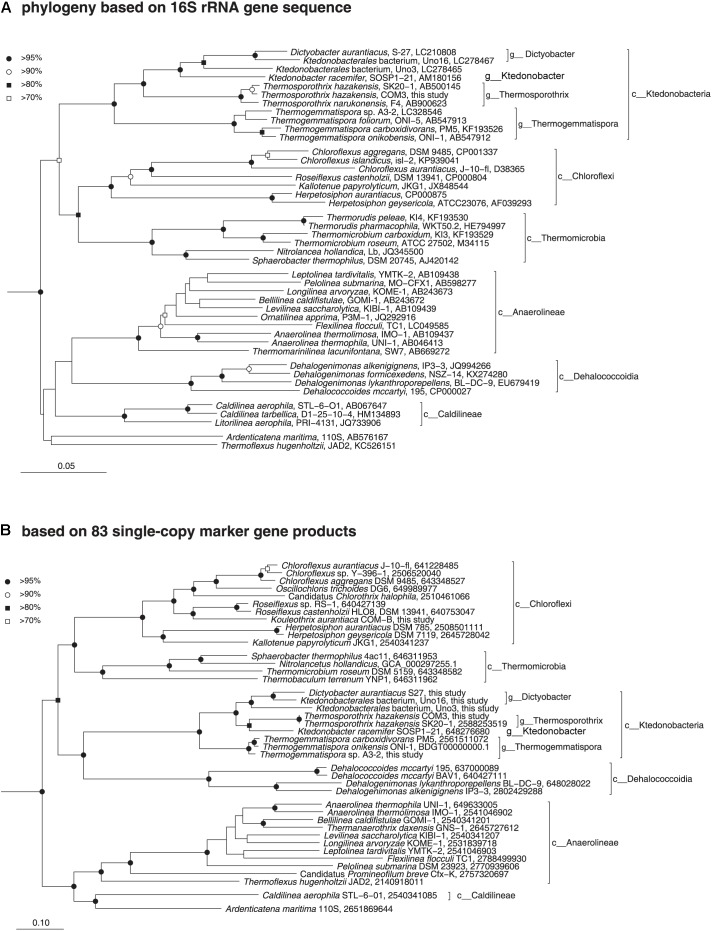
Phylogenetic positions of the members of the class *Ktedonobacteria* based on comparative analysis of 16S rRNA gene sequences **(A)** and conserved protein sequences **(B)**. **(A)** Neighbor-joining phylogenetic tree of public data (accession numbers are shown at the end of the index of each note) and the 16S rRNA gene sequence of *T. hazakensis* COM3, which was extracted from the genome sequence obtained in this study. Sequences from the bacterial phylum *Nitrospira* were used to root the tree (not shown). Reproducible nodes are marked based on bootstrap values from neighbor-joining and maximum-likelihood inferences (closed circle, >95% for both inferences; open circle, >90%; closed square, >80%; open square, >70%). Nodes that lack symbols were not reproducible among trees. Bar: 5% estimated sequence divergence. **(B)** Maximum-likelihood phylogenetic inference of representative genomes of the class *Chloroflexi*. The tree was built using RAxML based on up to 83 universally conserved proteins. Reproducible associations based on bootstrap values (closed circle, >95% for both RAxML and FastTree inferences; open circle, >90%; closed square, >80%; open square, >70%) are indicated at interior nodes. Accession numbers of genomes (IMG or DDBJ/EMBL/GenBank databases) are shown at the end of the index of each taxon. Alignments of homologous proteins from genomes of members of the phylum *Deinococci* were used to root the tree (not shown). Bar: 10% estimated sequence divergence.

Phylogenetic position of the class *Ktedonobacteria* was determined via a comprehensive analysis of 16S rRNA gene sequences and universally conserved protein sequences. Initially, a phylogenetic association between the class *Ktedonobacteria* and the phylum *Chloroflexi* was suspected owing to the low bootstrap values in the 16S rRNA gene sequence phylogenetic tree and the dissimilarities in morphological, physiological, and chemotaxonomic data between the two (Cavaletti et al., 2006). However, our phylogenetic analyses based on both 16S rRNA gene sequences (Figure 3A) and 38 and 83 single-copy marker genes (Figure 3B and Supplementary Figure S1) clearly inferred that the members of the class *Ktedonobacteria* belong to the phylum *Chloroflexi*. The genome-based phylogenies determined in this study added evidence supporting the idea that the members of the class *Ktedonobacteria* evolved from a common descendent of the phylum *Chloroflexi*, although they largely shared some important phenotypic traits with those of members of the phylum *Actinobacteria*. Notably, this is the first report of the formation of a monophyly between *Ktedonobacteria* and *Dehalococcoidetes*, another class in the phylum *Chloroflexi* and which is characterized with small genomes and obligate niche adaptation to reductive dehalogenation as the sole catabolic metabolism (Kaster et al., 2014).

Meanwhile, an obvious formation of a monophyly between strain *Ktedonobacterales* bacterium Uno16 and *D. aurantiacus* S27^T^ was observed in both 16S rRNA and genomic phylogenetic trees (Figure 3). The 16S rRNA genetic similarity and average nucleotide identity (ANI) between the two strains were 95.60% and 74.26%, respectively, suggesting strain *Ktedonobacterales* bacterium Uno16 could represent a novel species within the genus *Dictyobacter*. As for *Ktedonobacterales* bacterium Uno3, the most closely related strains were *D. aurantiacus* S27^T^ and *K. racemifer* SOSP1-21^T^, at the 16S rRNA genetic similarities of 91.46% and 89.98%, respectively. The average nucleotide identities of *Ktedonobacterales* bacterium Uno3 with *D. aurantiacus* S27^T^ and *K. racemifer* SOSP1-21^T^ were 69.86% and 69.70%, respectively. This suggested that *Ktedonobacterales* bacterium Uno3 belongs to an unclassified novel genus within the order *Ktedonobacterales*. The full taxonomic characteristics of Uno3, Uno16, and Uno17 were conducted in another study (Wang et al., 2019).

The pan-genome concept was firstly coined by Tettelin et al. (2005) in to catalog the entire genomic genes of a given phylogenetic clade, including core genes (detected in all strains), accessory genes (detected in two or more strains), and unique genes (strain-specific genes), and was also used to estimate the genetic diversity of a studied group. In this study, we performed the pan-genome analysis to compare the nine strains within class *Ktedonobacteria*, and with the class *Dehalococcoidetes*, which formed a monophyly with the class *Ktedonobacteria* on the 38 and 83 single-copy marker genes based phylogenetic trees (Figure 3B and Supplementary Figure S1). As summarized in Supplementary Table S5A, 81.00∼83.73% of the total genes were shared between strains *Ts. hazakensis* SK20-1^T^ and *Ts. hazakensis* COM3, while the numbers were 77.50∼77.80% in the genus *Thermogemmatispora*. By contrast, only 55.22∼54.64% of genes were shared between *D. aurantiacus* S27^T^ and *Ktedonobacterales* bacterium Uno16. Moreover, the percentages of core genes changed to 57.45∼63.26% (Supplementary Table S5B) and 32.48∼36.59% (Supplementary Table S5C), when contig1 (chromosome) and contig2 (“megaplasmid”) were aligned separately. Additionally, we found that *Ktedonobacterales* bacterium Uno3 shared the highest core gene ratio (38.84%) with the genus *Dictyobacter* among the class *Ktedonobacteria*. As for *K. racemifer* SOSP1-21^T^, the highest core gene ratio was found to be with the genus *Thermosporothrix*, at 21.06% of its total genes. The close relationships between *K. racemifer* SOSP1-21^T^ with the genus *Thermosporothrix*, and *Ktedonobacterales* bacterium Uno3 with the genus *Dictyobacter* were in accordance with the result of the above phylogenetic analyses based on 83 single-copy marker genes (Figure 3B). As for the class *Ktedonobacteria*, a total of 1181 core genes were shared among the nine strains, although they belonged to two orders and five genera. When the nine *Ktedonobacteria* strains were aligned with the class *Dehalococcoidetes*, we found the highest number of core genes (112 core genes) and highest core gene ratio (2.54∼2.59%) in the genus *Thermogemmatispora*. However, the entire core genes number (76 core genes) shared between the class *Ktedonobacteria* and the class *Dehalococcoidetes* was not large enough to overwhelm the number (69 core genes) shared with representative strains from other classes in the phylum *Chloroflexi* (Supplementary Table S5A).

**FIGURE 4:**
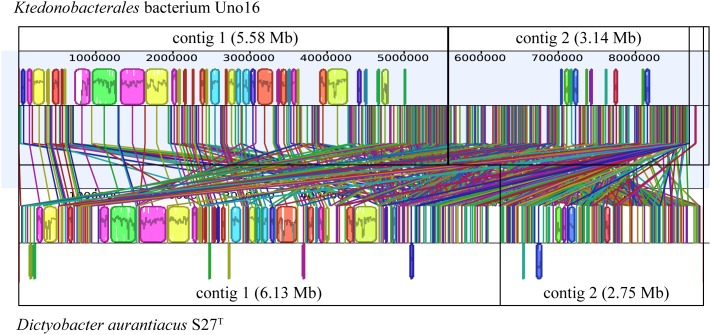
Comparative analyses of strains *D. aurantiacus* S27^T^ and *Ktedonobacterales* bacterium Uno16. The colored boxes represent homologous regions with another genome. Lines between two genomes trace each homologous region between them.

To compare the genomic rearrangement between *D. aurantiacus* S27^T^ and *Ktedonobacterales* bacterium Uno16, which were proposed to comprise putative “megaplasmids,” homologous regions in genomes were aligned between the two strains. As described above, strain *Ktedonobacterales* bacterium Uno16 was considered a novel species within the genus *Dictyobacter* by phylogenetic analyses based on both 16S rRNA gene sequences and universally conserved protein sequences. However, although genomic rearrangement in contig1 of strain *Ktedonobacterales* bacterium Uno16 was observed to be highly homologous to the contig1 of *D. aurantiacus* S27^T^, contig2 of the two strains were very dissimilar (Figure 4).

### Overview of the Putative Secondary Metabolites Biosynthetic Gene Clusters

**FIGURE 5:**
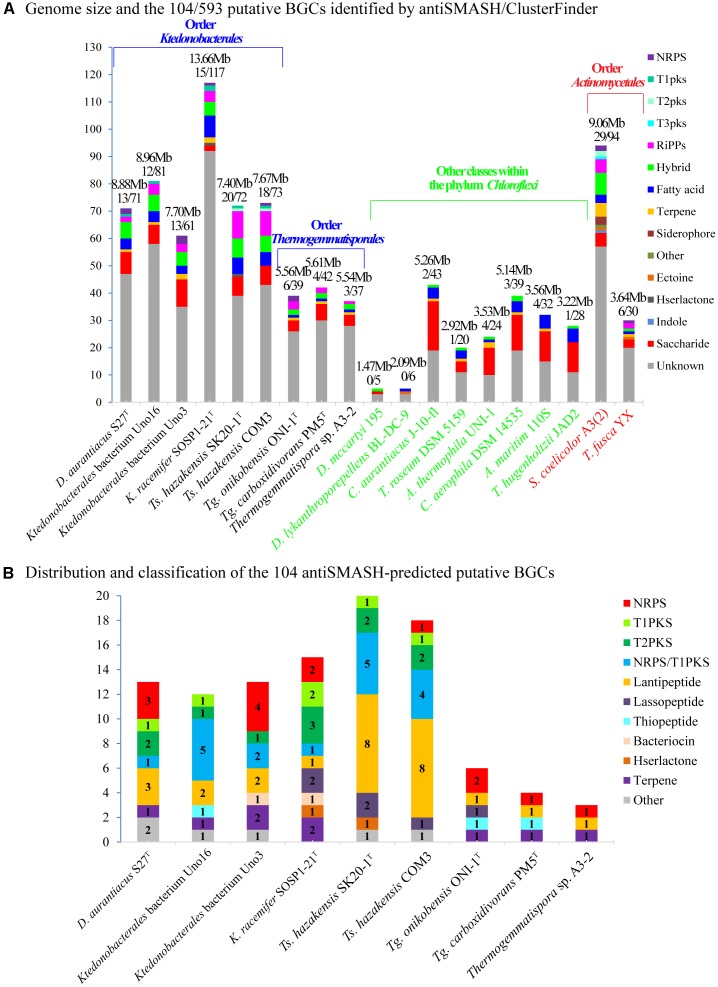
Composition and distribution of the ClusterFinder-identified putative BGCs **(A)** and antiSMASH-identified putative BGCs **(B)** in the nine *Ktedonobacteria* genomes. Strains *Dehalococcoides mccartyi* 195 and *Dehalogenimonas lykanthroporepellens* BL-DC-9 (class *Dehalococcoides*), *Chloroflexus aurantiacus* J-10-f1 (class *Chloroflexi*), *Thermomicrobium roseum* DSM 5159 (class *Thermomicrobia*), *Anaerolinea thermophila* UNI-1 (class *Anaerolineae*), *Caldilinea aerophila* DSM 14535 (class *Caldilineae*), *Ardenticatena maritima* strain 110S (class *Ardenticatenia*), and *Thermoflexus hugenholtzii* JAD2 (class *Thermoflexia*) of the phylum *Chloroflexi* and strains *Streptomyces coelicolor* A3(2) and *Thermobifida fusca* YX within order *Actinomycetales*, phylum *Actinobacteria* were used as reference strains for comparison.

To evaluate the biosynthetic potential of the class *Ktedonobacteria*, antiSMASH version 4.2.0 plus the integrated ClusterFinder algorithm were used to predict both characterized and unknown functioned secondary metabolite BGCs in the nine genomes. As shown in Figure 5A and Supplementary Table S6A, a total of 593 putative characterized and unknown BGCs were predicted by ClusterFinder, with 37∼117 BGCs per genome and counting for 11.75∼34.49% of the genomic sequences. To gain a deeper understanding of the *Ktedonobacteria* secondary metabolites, we focused on the putative BGCs with characterized functions predicted by antiSMASH. Initially, 104 antiSMASH-identified putative BGCs were found in the nine *Ktedonobacteria* genomes, including 14 NRPS clusters, 17 PKS clusters, 18 hybrid PKS/NRPS clusters, 27 lantipeptide clusters, and 28 other BGCs (Figure 5B and Supplementary Table S6B). However, these BGCs were distributed very unevenly in the nine strains. Strain *Ts. hazakensis* SK20-1^T^ and *Ts. hazakensis* COM3 encoded 20 and 18 BGCs, dedicating 15.28% and 12.64% of their total genome, respectively (Supplementary Table S6B). In contrast, an average of four BGCs were identified in the genome of *Thermogemmatispora* strains, constituting only 1.78∼2.48% of the total genome on average. Strains *D. aurantiacus* S27^T^, *Ktedonobacterales* bacterium Uno16, and *Ktedonobacterales* bacterium Uno3 encoded 13, 12, and 13 antiSMASH-identified BGCs, dedicating 6.46%, 7.54%, and 12.89% of their total genomes, respectively. In accordance with the result of ClusterFinder, 35.27% of the genomic sequences on contig 2 of *Ktedonobacterales* bacterium Uno3 were predicted to encode antiSMASH-identified putative BGCs. Moreover, composition of the antiSMASH-identified putative BGC types were very diverse in the nine *Ktedonobacteria* strains. The main BGC types in *Ts. hazakensis* SK20-1^T^ and *Ts. hazakensis* COM3 were NRPS/T1PKS hybrid BGCs and lantipeptide BGCs, while *D. aurantiacus* S27^T^, *Ktedonobacterales* bacterium Uno3, and *K. racemifer* SOSP1-21^T^ were more abundant in NRPS and PKS BGCs. Unlike *D. aurantiacus* S27^T^, *Ktedonobacterales* bacterium Uno16 encoded more Lantipeptide and NRPS/T1PKS hybrid BGCs on its genome. Overall, it was observed that BGCs responsible for peptide compounds production including NRPS, lantipeptide, lassopeptide, and thiopeptide BGCs predominate in the nine *Ktedonobacteria* genomes, while PKS BGCs encoding the polyketide compounds were the second most abundant BGC types. Herein, we analyzed composition and organization of the *Ktedonobacteria* NRPS, PKS, NRPS/PKS hybrid, and lantipeptide BGCs in the next section. In addition, the 104 antiSMASH-identified BGCs exhibited very limited similarity with known clusters in the MIBiG database (Supplementary Table S6B).

### NRPS, PKS, NRPS/PKS Hybrid, and Lantipeptide BGCs

A total of 14 putative antiSMASH-identified NRPS BGCs were identified albeit distributed unevenly in seven strains, ranging from 20.4 kb (cluster Toni_5 in strain *Tg. onikobensis* ONI-1^T^) to 154.4 kb (cluster Uno3_7 in strain *Ktedonobacterales* bacterium Uno3) in size. Domain composition and organization analysis (Supplementary Table S7A) indicated that some of the NRPS BGCs such as cluster Txc_10 and cluster Dar_12 could produce peptide products with a ring moiety, by the existence of multiple epimerization (E) domain and heterocyclization (HC) domain (Chen L.Y. et al., 2016; Bloudoff et al., 2017) in the clusters. In addition, the existence of fatty acyl-AMP ligase (FAAL) domain in cluster Uno3_5 suggests that the final product of this gene cluster may constitute a lipopeptide or its derivatives. The FAAL domain was first discovered in *Mycobacterium tuberculosis* and activates fatty acids such as acyl adenylates, subsequently catalyzing their transfer onto the ACPs of PKSs or non-ribosomal peptide synthetases to produce lipidic metabolites (Hayashi et al., 2011).

As for PKS BGCs, 6 T1PKS BGCs and 11 T2PKS BGCs were identified. The 6 T1PKS BGCs spanned from 34.2 kb (cluster Krac_15 in strain *K. racemifer* SOSP1-21^T^) to 46.6 kb (cluster Uno16_10 in strain *Ktedonobacterales* bacterium Uno16) in size whereas the 11 T2PKS BGCs spanned from 41.3 kb (cluster Uno16_1 in strain *Ktedonobacterales* bacterium Uno16) to 42.5 kb (cluster Krac_11 in strain *K. racemifer* SOSP1-21^T^). Domain composition and organization of the identified PKS clusters are summarized in Supplementary Tables S7B,C. Notably, cluster Uno16_10 contained two additional NRPS-A domains that were predicted to function as a long chain fatty acid CoA ligase and an acyl CoA synthetase by Protein-Protein Blast, indicating that the final products of this cluster may comprise derivatives of lipids (Hisanaga et al., 2004; Soupene and Kuypers, 2008).

Eighteen putative antiSMASH-identified BGCs were classified to be NRPS-T1PKS hybrid clusters, which were distributed mainly in strains *Ts. hazakensis* SK20-1^T^, *Ts. hazakensis* COM3, and *Ktedonobacterales* bacterium Uno16. The 18 putative hybrid NRPS-T1PKS clusters ranged in size from 54.4 kb (cluster Uno16_4 in strain *Ktedonobacterales* bacterium Uno16) to 333.5 kb (cluster Uno3_11 in strain *Ktedonobacterales* bacterium Uno3). Domain composition and organization and the AMP-binding domain amino acid substrate specificities of the hybrid clusters are summarized in Supplementary Tables S7D,E. Based on genetic similarity and domain composition and organization in these gene clusters, the 18 putative hybrid NRPS-T1PKS clusters could be divided into four groups. Moreover, the hybrid NRPSs and PKSs biosynthesis pathways obviously enlarge the diversity of bacterial natural products.

After a comprehensive analysis of antiSMASH and BAGEL3 (van Heel et al., 2013), the 27 lantipeptide BGCs were further classified into four classes according to their biosynthetic machinery (Zhang et al., 2012). Class I contains 11 lantipeptide BGCs that are synthesized by two separated modification genes, *LanB* and *LanC*. In addition, cluster Txs_13 from strain *Ts. hazakensis* SK20-1^T^ comprised two *LanB* genes and one *LanC* gene. However, the two *LanB* genes were short in size and both lacked a lantibiotic biosynthesis dehydratase C-terminal domain, according to Pfam analysis. As the C-terminal domain is necessary for the final glutamate-elimination step in the generation of lantipeptide (Ortega et al., 2015), we propose these two *LanB* genes may be inactive. Class II contains 11 lantipeptide BGCs that are synthesized by a single bifunctional enzyme termed LanM. Moreover, after conducting a ClustalW alignment using MEGA7.0 and generating sequence logos with WebLogo 3 (Crooks et al., 2004), we observed F(E/D)LD (Supplementary Figure S2A) and GG (Supplementary Figure S2B) cleavage sites in class I and class II, respectively.

Based on these analyses, the antiSMASH-identified *Ktedonobacteria* BGCs showed very limited homology with known BGCs. The different composition and organization of tailoring genes, transport-related genes, and regulatory genes further increased the diversity of these BGCs and indicated that these clusters may encode novel natural products with novel functions.

### Phylogenetic Analysis of the PKS KS Domain, NRPS C Domain, and Lantipeptide Modification Genes

**FIGURE 6:**
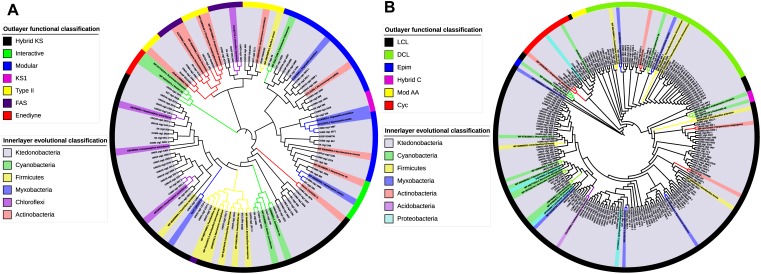
Functional and evolutionary analysis of *Ktedonobacteria* PKS KS and NRPS C domains. **(A)** Domain-specific phylogenetic analysis of the *Ktedonobacteria* amino acid sequences extracted from KS domains. The reference sequences include three top hits from Protein-Protein Blast and the NaPDoS reference database. Maximum-likelihood phylogenetic tree of the *Ktedonobacteria* KS domains was built in MEGA v. 7.0. The evolutionary classification was shown in the inner layer color strip whereas the outer layer color strip represents the functional classification of the KS domains. **(B)** Domain-specific phylogenetic analysis of the *Ktedonobacteria* amino acid sequences extracted from C domains. The analysis was created as for **(A)**.

Considering that class *Ktedonobacteria* constitutes a relatively new bacterial taxa, to date only very limited knowledge is available regarding their secondary metabolites. The domain-specific phylogenetic analysis of *Ktedonobacteria*-originated secondary metabolite BGCs identified in the present study may provide a better understanding of the functional and evolutionary classification of their domains. Furthermore, as C and KS domains are responsible for peptide and polyketide chain elongation in NRPS and PKS biosynthesis, respectively, the two domains represent the best candidates for domain-specific phylogenetic analysis (Rausch et al., 2007; Jenke-Kodama and Dittmann, 2009). As shown in the outlying ring of Figure 6A, the most abundant functional type among the *Ktedonobacteria* KS domains was assigned to hybrid KS by NaPDoS classification, owing to the high occurrence of hybrid NRPS-T1PKS clusters identified in the *Ktedonobacteria* genomes. Modular KS, the second most abundant KS domain functional type in the class *Ktedonobacteria*, was also mainly derived from hybrid NRPS-T1PKS clusters. The modular PKS is responsible for the incorporation of one building block and contains at least three domains: KS, AT, and ACP (Jenke-Kodama et al., 2005). With regard to evolutionary classification, the *Ktedonobacteria*-originated KS domains and reference sequences appeared to form four clades. The first clade included a sequence from strain *Ktedonobacterales* bacterium Uno3, a sequence from strain *D. aurantiacus* S27^T^, and two sequences derived from the phylum *Cyanobacteria*.

The other three clades were more diverse in representation, including sequences from the phyla *Actinobacteria, Cyanobacteria, Firmicutes, Chloroflexi*, and the order *Myxobacteria*. Moreover, part of the *Ktedonobacteria* KS domains clustered with *Actinobacteria, Cyanobacteria*, and *Firmicutes* reference sequences, indicating that they may be phylogenetically related to these phyla. However, the majority of the *Ktedonobacteria*-originated KS domains formed independent branches. Furthermore, the highest similarity between *Ktedonobacteria* KS domains and reference sequences was only 71%, as observed between cluster Uno3_11 and a *Brevibacillus laterosporus* reference sequence. According to the NaPDoS functional classification, the most abundant types of *Ktedonobacteria*-originated C domains are LCL and DCL. The LCL type C domain catalyzes formation of a peptide bond between two L-amino acids whereas the DCL type links an L-amino acid to a growing peptide ending with a D-amino acid in NRPS biosynthesis (Rausch et al., 2007). In agreement with our analysis of NRPS and hybrid clusters, the C domains, which are replaced by HC domains in strain SK20-1^T^ and strain COM3, were classified as cyclization domains by NaPDoS, which catalyze both peptide bond formation and subsequent cyclization of cysteine, serine, or threonine residues. With regard to evolutionary classification, the *Ktedonobacteria* C domains showed more diversity compared with the KS domains. As shown in Figure 6B, only a small percentage of C domains were related to the reference sequences from *Cyanobacteria, Actinobacteria, Firmicutes, Myxobacteria, Proteobacteria*, and *Acidobacteria*. Rather, a large proportion of *Ktedonobacteria* C domains formed independent branches, indicating they are distinct from those that originated from other phyla in evolutionary taxonomy.

As modification genes are responsible for the maturation of lantipeptide, the phylogeny of these modification enzymes are closely correlated with the structure of final lantibiotic natural products (Willey and van der Donk, 2007; Zhang et al., 2012). In the present study, the phylogenetic analyses were also applied to *Ktedonobacteria* modification genes to determine their evolutionary relationship with other bacterial phyla or classes. As confirmed in the phylogenetic tree (Supplementary Figure S3), the majority of the *Ktedonobacteria* LanB and LanC modification genes formed independent clusters although they showed limited similarities with reference sequences from the phylum *Actinobacteria*. Compared with LanB and LanC modification genes, greater diversity was observed among LanM genes (Supplementary Figure S3). The two LanM genes from cluster Dar_6 and the LanMM cluster in strain S27^T^ were related to LanMs from the phylum *Cyanobacteria*. The other *Ktedonobacteria* LanMs showed similarities with *Actinobacteria* and *Myxobacteria* sequences, whereas the majority formed independent branches, indicating that they may modify structurally different products from the known lantibiotic natural products.

### Antimicrobial Activities of *Ktedonobacteria*

Cell pellets and HP-20 polyaromatic adsorbent resin crude extract from six representative *Ktedonobacteria* strains were tested for antimicrobial activities against both Gram-positive and Gram-negative bacterial strains. All six *Ktedonobacteria* strains demonstrated activity against at least two of the nine tested bacterial strains (Table 4). In addition, all six *Ktedonobacteria* strains were positive against *G. stearothermophilus* NBRC 13737, *S. aureus* NBRC 13276, and *S. aureus* Mu50.

**Table 4:** Antimicrobial activity of the representative *Ktedonobacteria* strains assessed using the paper disc method.

Strain	Antimicrobial activity (zone of inhibition in mm)								

	1	2	3	4	5	6	7	8	9
*D. aurantiacus* S27^T^	11.2	10.0	15.0	9.0	18.5	18.5	13.0	16.0	21.0
*Ktedonobacterales* bacterium Uno16	8.4	10.0	11.5	9.0	21.5	21.5	10.0	15.0	20.0
*Ktedonobacterales* bacterium Uno3	–	9.0	11.5	9.5	22.0	22.0	8.4	13.5	16.0
*K. racemifer* SOSP1-21^T^	–	–	–	–	–	–	7.5	–	7.5
*Ts. hazakensis* COM3	7.0	–	–	–	21.0	21.0	7.5	9.8	14.5
*Thermogemmatispora* sp. A3-2	–	–	–	–	10.0	10.0	–	–	7.8


*1. *B. subtilis* NBRC 3134; 2. *P. aeruginosa* NBRC 13275; 3. *E. coli* NBRC 3972; 4. *S. enterica* NBRC 100797; 5. *M. luteus* NBRC 13867; 6. *G. stearothermophilus* NBRC 13737; 7. *S. aureus* NBRC 13276; 8. *S. aureus* NTCT8325 (MSSA); 9. *S. aureus* Mu50 (VISA).*

Overall, the three mesophilic *Ktedonobacteria* strains including *D. aurantiacus* S27^T^, *Ktedonobacterales* bacterium Uno3, and *Ktedonobacterales* bacterium Uno16, exhibited a broader antibacterial spectra against both Gram-positive and Gram-negative (*P. aeruginosa* NBRC 13275, *E. coli* NBRC 3972, and *S. enterica* NBRC 100797) bacterial strains. In comparison, strain *Ts. hazakensis* COM3 and *Thermogemmatispora* sp. A3-2, the two thermophilic strains, only inhibited the Gram-positive bacterial strains (*M. luteus* NBRC 13867, *G. stearothermophilus* NBRC 13737, and *S. aureus* Mu50). Moreover, the three mesophilic *Ktedonobacteria* strains also exhibited stronger inhibitory capacities against each Gram-positive bacterial strains than that shown by the two thermophilic strains, as evidenced by the formation of larger inhibition clear zones (Table 4 and Supplementary Figure S4).

## Discussion

### Phylogenetic and Evolutional Analysis

Members of the phylum *Chloroflexi*, especially the class *Chloroflexi*, are generally known as filamentous anoxygenic phototrophs, also termed filamentous green non-sulfur bacteria (Björnsson et al., 2002). However, the phylum *Chloroflexi* also constitutes a deeply branched bacterial lineage encompassing classes of bacteria with diverse metabolic types from strictly anaerobic chlorinated hydrocarbon reducers to filamentous aerobic heterotrophs and vastly distinct genome sizes from 1.38 to 13.67 Mb (Cavaletti et al., 2006; Moe et al., 2009; Chang et al., 2011; Kaster et al., 2014). Moreover, comparative genomics and phylogenetic analyses suggest that these different classes may represent related but distinct phyla under a *Chloroflexi* “superphylum” (Gupta et al., 2013). Notably, maximum likelihood phylogenomic analysis based on informative amino acid sequences confirmed the class *Ktedonobacteria* and the SAR202 clade of dark-ocean bacterioplankton as the two deepest-branching classes related to the common ancestor of the phylum *Chloroflexi* (Landry et al., 2017). As the phylum *Chloroflexi* constitutes one of the earliest bacterial branches in the terrestrial environment (Hug et al., 2016), the class *Ktedonobacteria* is therefore considered to be among the earliest diverging bacterial lineages.

In the phylogenetic tree based on conserved protein sequences, class *Ktedonobacteria* clustered together with *Dehalococcoidetes*, a class of a strictly anaerobic, slow growing, and highly niche-specialized bacteria in terrestrial aquifer environments that utilize organohalide respiration as their sole source of energy (Kaster et al., 2014). However, our pan-genome analysis between class *Ktedonobacteria* and class *Dehalococcoidetes* indicated the two bacterial classes do not share a high number of conserved core genes with each other. Moreover, the two classes are clearly distinct in morphologies, genome sizes, habitats, and metabolic styles. Nonetheless, the formation of a monophyly in the phylogenetic tree based on conserved protein sequences suggest they may have evolved from a common ancestor. Herein, we hypothesize that the two bacterial classes then evolved separately to adapt to different environments and the evolutionary results reflect on their genomes. The class *Dehalococcoidetes* evolved a sole energy conservation mode via organohalide respiration and developed a symbiotic metabolic style, depending on co-living microbial species to acquire electron donors and cofactors (Kaster et al., 2014). As a result, species in the class *Dehalococcoidetes* may gradually drop unnecessary genes in their genomes, resulting in small genome sizes. On the contrary, species in the class *Ktedonobacteria* are very diverse in habitats, ranging from common soil to oligotrophic environments. Thus, species in the class *Ktedonobacteria* may tend to obtain foreign genes or foreign secondary metabolites gene clusters to adapt to these environments, resulting in relatively large genomes. Moreover, the class *Ktedonobacteria* developed a spore-forming morphology to survive nutrient depletion or harsh environments (Errington, 2003; Flärdh and Buttner, 2009). Of course, the currently sequenced genomes of the phylum *Chloroflexi* are still very limited and further studies are needed to completely characterize the evolution of class *Ktedonobacteria*.

### Genome Features of the Class *Ktedonobacteria*

As described above, species in the class *Ktedonobacteria* are featured with low GC content and relatively large genome sizes, among which genomes of strains *D. aurantiacus* S27^T^, *Ktedonobacterales* bacterium Uno16, *Ktedonobacterales* bacterium Uno3, and *K. racemifer* SOSP1-21^T^ are comparable to that of actinomycetes strains (Chen W.H. et al., 2016). As for the studies of strains in genus *Thermosporothrix* and genus *Thermogemmatispora*, their genome sizes are also quite large among thermophilic bacteria given that growth temperature and genome size in bacteria are negatively correlated and thermophilic bacteria tend to have a small genome (Sabath et al., 2013). Also, it has been reported that the *Firmicutes* and *Gammaproteobacteria* have the largest average 16S rRNA gene copy numbers (6.788 ± 2.694 and 5.141 ± 2.411, respectively) and the maximum variation (5.458 ± 5.633 and 5.298 ± 7.052, respectively) (Ibal et al., 2019). Thus, the average copy numbers of 16S rRNA genes (6.375 per genome, excluding strain *Tg. onikobensis* ONI-1^T^) in *Ktedonobacteria* genomes is quite high in bacterial species, although their variations are lower than *Firmicutes* and *Gammaproteobacteria*. Moreover, the relatively high percentage of functional unknown hypothetical proteins indicated that the class *Ktedonobacteria* may possess as yet unknown metabolites and produce some novel natural products through novel mechanisms (Galperin, 2001).

It is interesting to find that strains *D. aurantiacus* S27^T^, *Ktedonobacterales* bacterium Uno16, and *Ktedonobacterales* bacterium Uno3 may possess “megaplasmids.” As described in Section “Strain Information and Whole Genome Sequencing,” the draft genome of *Ktedonobacterales* bacterium Uno3 includes two circular contigs (Figure 2). As the presence of housekeeping genes are essential to define a bacterial chromosome (Thomas and Summers, 2008), herein, it is probably more appropriate to classify the second circular contig of Uno3 to be “megaplasmid” due to the absence of most housekeeping genes (Table 3 and Supplementary Table S2). The presence of megaplasmid was also observed in *Thermomicrobium roseum* DSM 5159, an aerobic CO oxidizing thermophile strain in the phylum *Chloroflexi* and composed one chromosome (2,006,217 bp) and one megaplasmid (919,596 bp) in which few standard housekeeping genes were found (Wu et al., 2009). As for the linear contig2 of strains *D. aurantiacus* S27^T^ and *Ktedonobacterales* bacterium Uno16, the definition of “megaplasmid” could be further evidenced with the absence of most housekeeping genes, translation genes, DNA replication and repair genes, and genes involved in TCA cycle and oxidative phosphorylation (Table 3), which are essential genes for growth. Furthermore, the high dissimilarities in sequences observed between contig2 of the two strains (Figure 4) indicated they originated differently. Meanwhile, high numbers of transporter related genes, cytochrome P450 related genes, and regulator genes were identified in contig2 of the two strains (Table 3). Commonly, these genes in bacterial genomes could contribute to their adaptation or resistance to various environmental pressures such as antibiotics, heavy metals, extreme Antarctic environments, among others (Kelly and Kelly, 2013; Dziewit et al., 2015; Romaniuk et al., 2018). Thus, it could be that strains *D. aurantiacus* S27^T^ and *Ktedonobacterales* bacterium Uno16 acquired the foreign “megaplasmids” naturally to adapt to various environments. Additionally, we found that housekeeping genes are also absent in eight contigs of SOSP1-21^T^ (Supplementary Table S2). Nonetheless, this hypothesis of “megaplasmids” needs to be further confirmed because of their large sizes.

Unlike actinomycetes, which are saprophytic bacteria, the class *Ktedonobacteria* is reported to be predominate in the oligotrophic habitats (Northup et al., 2011; Stres et al., 2013; de Miera et al., 2014b; Tebo et al., 2015; Jiang et al., 2016) although they were also ubiquitous in various common terrestrial environments (Cavaletti et al., 2006; Yabe et al., 2017b). Additionally, the abundance of *Ktedonobacteria* was reported to increase or predominate in the bacterial communities with increasing CO_2_ flux in CO_2_ gas vents, CO_2_-rich hydrothermal spring and soil environments (de Miera et al., 2014a,b; Arce-Rodríguez et al., 2019). Together with our finding of the almost complete reductive TCA cycle and the presence of multiple copies of *cox* genes in genomes, it is highly possible that the class *Ktedonobacteria* may possess unknown autotrophic carbon fixation pathways.

### Secondary Metabolites Biosynthetic Potential of the Class *Ktedonobacteria*

In this study, a total of 593 ClusterFinder-identified putative BGCs were predicted in nine *Ktedonobacteria* strains among which 104 putative BGCs were also predicted by antiSMASH. As the genome size of bacteria is considered to correlate with the number of gene clusters (Donadio et al., 2007), the high number of putative BGCs predicted could be a consequence of their large genomes. Moreover, we observed that the class *Ktedonobacteria* encode numerous NRPS, PKS, NRPS/PKS hybrid, and lantipeptide gene clusters, which may assist them in fighting against their competitors and predators in their niches (Challis and Naismith, 2004; Zhang et al., 2012; Johnston et al., 2015; Ortega and van der Donk, 2016; Naughton et al., 2017). This hypothesis could be supported by the observation that the genus *Thermogemmatispora*, which exhibited the lowest number of BGCs in the class *Ktedonobacteria*, predominates in geothermal niches in which the microbial community richness is markedly decreased (de Miera et al., 2014b; Jiang et al., 2016). Owing to a lack of competitors and predators (Yabe et al., 2017a), the genus *Thermogemmatispora* may tend to reduce the number of BGCs in their genomes as secondary metabolites are not necessary but are always costly for microorganisms. In addition, the majority of the 104 antiSMASH-identified putative BGCs showed no or very limited similarities with known clusters. This result could also be supported by the functional and evolutionary phylogenetic analysis of the *Ktedonobacteria* KS and C domains. As shown in Figure 6, the majority of *Ktedonobacteria*-derived KS and C domains formed independent clusters from those derived from other phyla.

Nevertheless, 38 out of the total 104 antiSMASH-identified putative *Ktedonobacteria* BGCs exhibited varying degrees of similarity to known gene clusters. As described in the Section “Results” and summarized in Supplementary Table S6, the 38 *Ktedonobacteria* BGCs were related to *Actinobacteria* (17 *Ktedonobacteria* BGCs), *Cyanobacteria* (14 *Ktedonobacteria* BGCs), *Myxobacteria* (4 *Ktedonobacteria* BGCs), *Firmicutes* (2 *Ktedonobacteria* BGCs), and *Gammaproteobacteria* (1 *Ktedonobacteria* BGCs) pathways. In addition, we observed that the 38 BGCs comprised mainly hybrid NRPS-T1PKS clusters (15 BGCs), NRPS clusters (7 BGCs), PKS clusters (7 BGCs), and RiPP clusters (7 BGCs). As aforementioned, these types of BGCs usually encode modular enzymes (Challis and Naismith, 2004; Dutta et al., 2014; Letzel et al., 2014; Ortega and van der Donk, 2016). Thus, the homolog of a single gene or single domain could result in the high similarity between two BGCs, which could be observed between the *Ktedonobacteria* hybrid PKS/NRPS cluster and *Myxobacterium S. cellulosum* pellasoren A gene cluster. The repetition of a KS-AT-cMT-KR-ACP PKS module-encoding gene in the pellasoren A gene cluster resulted in the 83% similarity between DNA sequences of the two gene clusters. Furthermore, the existence of a homologous CAL domain in cluster Uno16_7 and the puwainaphycins BGC resulted in a 20% genetic similarity between the two clusters.

However, as few descriptions regarding secondary metabolites of the class *Ktedonobacteria* are available, the limited similarities between BGCs from the class *Ktedonobacteria* and those from other bacterial phyla may contribute to our understanding of the biosynthetic pathways of secondary metabolites in *Ktedonobacteria*. Moreover, the majority of the *Ktedonobacteria* BGCs are totally novel and differ from the known BGCs with regard to composition and organization of the condensation domains in NRPS BGCs, ketosynthase domains in PKS BGCs, and leader peptide in lantipeptide clusters. In addition, the difference of transport-related genes and regulatory genes with known BGCs further increases the diversity of known *Ktedonobacteria* BGCs. Notably, the BGC similarities revealed by antiSMASH comparison and the results of *Ktedonobacteria* ketosynthase and condensation domain phylogenetic analysis (Figure 6) suggest that a portion of the secondary metabolite BGCs in the class *Ktedonobacteria* may have been acquired from other bacterial phyla via horizontal gene transfer (HGT). HGT is common in bacteria and may provide new functions and subsequent niche adaptations through the acquisition of valuable secondary metabolite BGCs (Tooming-Klunderud et al., 2013; Ziemert et al., 2014; Jensen, 2016; Adamek et al., 2018). BGCs encoding natural products usually include both core biosynthetic and tailoring enzymes, along with regulatory genes for biosynthesis, resistance genes to the natural products they produce, and transport genes such as efflux pumps to export these natural products from their cells to the extracellular environment (Jensen, 2016). In addition, the regulatory genes required for function may facilitate integration of the HGT-acquired genes into existing genomes (Lawrence, 1999). Thus, the existence of regulatory, resistance, and transport genes in NRPS, PKS, and their hybrid gene clusters might facilitate their HGT (Ziemert et al., 2014). However, as the class *Ktedonobacteria* is among the earliest diverging bacterial lineages, arising much earlier than the phyla *Actinobacteria, Cyanobacteria, Firmicutes*, and *Proteobacteria* (Hug et al., 2016; Landry et al., 2017), we cannot exclude the possibility that the class *Ktedonobacteria* constitutes the actual HGT origin of secondary metabolite gene clusters.

The representative *Ktedonobacteria* strains produced probable bioactive compounds against both Gram-positive and Gram-negative bacterial strains (Table 4 and Supplementary Figure S4), suggesting their potential utility for antimicrobial screening. Among the six screened *Ktedonobacteria* strains, *D. aurantiacus* S27^T^, *Ktedonobacterales* bacterium Uno3, and Uno16 were the most capable. Given that Gram-negative bacterial strains are becoming increasingly antibiotic resistant owing to their protective outer membranes and constitutively active efflux pumps, these mesophilic *Ktedonobacteria* strains may contribute to the development of novel antibiotics targeting Gram-negative bacteria (Miller, 2016; Domalaon et al., 2018). In turn, the antibacterial activities against *S. aureus* NBRC 13276, *S. aureus* NTCT8325, and *S. aureus* Mu50 might be explained by the production of novel lanthipeptides or other antimicrobial peptides by the *Ktedonobacteria* strains, as reported previously (Xu et al., 2018). Moreover, as the candidate testing microorganisms used for antimicrobial assays and activities assessed in the present study were finite and few isolated *Ktedonobacteria* species are available for study, this class likely harbors as-yet undisclosed bioactive functions.

## Conclusion

In this study, we performed whole genome sequencing, comprehensive phylogenetic analysis, genome comparisons, and biosynthetic potential analysis of the class *Ktedonobacteria*, one of the deepest-branching classes related to the common ancestor of the earliest diverging bacterial lineages *Chloroflexi*. Our results further confirmed the classification of the spore-forming *Ktedonobacteria* to the phylum *Chloroflexi* at both the 16S rRNA and genomic level. The existence of a putative “megaplasmid” in some of the *Ktedonobacteria* strains was also observed in this study. Meanwhile, we found the class *Ktedonobacteria* were characterized with relatively large genome sizes, multiple copies of ribosomal RNA operons, and a high ratio of hypothetical proteins with unknown functions. The possibilities of reductive TCA cycle were also observed in the class *Ktedonobacteria.* Analysis of putative secondary metabolites BGCs in the nine *Ktedonobacteria* strains yielded a total of 593 ClusterFinder-identified putative BGCs (104 antiSMASH-identified putative BGCs), representing the first study to mine the biosynthetic potential of the class. The overall novelty and diversity of these BGCs provided convincing evidence that the class *Ktedonobacteria* possesses broad potential to produce new metabolite products with novel structures and mechanisms. Furthermore, bioactivity screening of the studied nine *Ktedonobacteria* strains revealed a wide spectrum of antibacterial activities. However, it should be noted that the current understanding of the *Ktedonobacteria* secondary metabolite pathways is still very limited, and their relationship with pathways of other bacterial phyla remains to be elucidated. Given the potential ability to produce novel and diverse bioactive natural products, we consider the ancient, ubiquitous, and mycelia-forming *Ktedonobacteria* to represent a versatile and promising microbial resource for pharmaceutical and biotechnological use.

## Author Contributions

YZ and SY conceived and designed the study. YZ, AS, and YM performed the experiments. YZ, AT, YuS, YaS, SY, C-MW, KU, HT, KA, and AY analyzed the results and data. YZ wrote the manuscript. All authors have read and approved the manuscript, and reviewed and confirmed the manuscript for publication.

## Conflict of Interest Statement

The authors declare that the research was conducted in the absence of any commercial or financial relationships that could be construed as a potential conflict of interest.

## Footnotes

1 https://smrt-analysis.readthedocs.io/en/latest/

2 https://www.pacb.com/training/hgap4-de-novo-assembly-application/

3 https://github.com/dparks1134/CommunityM

4 https://blast.ncbi.nlm.nih.gov/Blast.cgi

5 https://www.megasoftware.net/

## Supplementary Material

FIGURE S1 Maximum-likelihood phylogenetic inference of members of the class *Ktedonobacteria* among known bacterial phyla. The tree was constructed using RAxML or FastTree based on up to 38 marker genes (using taxon-outgroup configuration Config 3 described in Sekiguchi et al., 2015) and sequences were collapsed at the phylum level except for classes in the *Proteobacteria*. Ranks are indicated by prefix; p_ (phylum), c_ (class). Parameters used for phylogenetic inference using RAxML and FastTree are described in the “Materials and Methods” section. Numbers at the notes indicate bootstrap values (%) with 100 times determination. The scale bar represents 10% estimated sequence divergence.

FIGURE S2 Sequence logos of precursor peptide conserved motifs identified in the nine *Ktedonobacteria* strains are underlined in red. **(A)** The “F(E/D)L” cleavage site for Class I lantipeptide gene clusters. **(B)** The “GG” cleavage site for Class II lantipeptide clusters.

FIGURE S3 Phylogenetic analysis of the *Ktedonobacteria* modification genes in lantipeptide clusters. **(A)** Phylogenetic analysis of the *Ktedonobacteria* LanB genes. Amino acid sequences extracted from the *Ktedonobacteria* LanB and the three top hit reference sequences from Protein-Protein Blast were aligned by MEGA v. 7.0. Maximum-likelihood method was used to build the tree. The background colors represent evolutionary classification of the LanB modification genes. **(B)** Phylogenetic analysis of the *Ktedonobacteria* LanC genes. The tree was built as for **(A)**. **(C)** Phylogenetic analysis of the *Ktedonobacteria* LanM genes. The tree was also built as for **(A)**.

FIGURE S4 Antimicrobial screening of six representative *Ktedonobacteria* strains against bacterial and fungal strains. The antimicrobial activity are indicated by formation of clear zone of *S. aureus* NTCT8325 (MSSA) **(A)**, *S. aureus* Mu50 (VISA) **(B)**, *Escherichia coli* NBRC3972 **(C)**, *Bacillus subtilis* NBRC3134 **(D)**, *Staphylococcus aureus* NBRC13276 **(E)**, *Geobacillus stearothermophilus* NBRC13737 **(F)**, *Pseudomonas aeruginosa* NBRC13275 **(G)**, *Salmonella enterica* NBRC100797 **(H)**, *Micrococcus luteus* NBRC13867 **(I)** as inhibited by *Thermosporothrix hazakensis* COM3 **(1)**, *Ktedonobacter racemifer* SOSP1-21^T^ **(2)**, *Dictyobacter aurantiacus* S27^T^ **(3)**, *Ktedonobacterales* bacterium Uno3 **(4)**, *Ktedonobacterales* bacterium Uno16 **(5)**, *Thermogemmatispora* sp. A3-2 **(6)**, and negative control **(N)**.

TABLE S1 Detailed sequencing information and genome features of the nine studied *Ktedonobacteria* strains and related taxon.

TABLE S2 CheckM estimation of the nine studied *Ktedonobacteria* strains.

TABLE S3 Similarities of multiple 16S ribosomal RNA operons within the nine studied *Ktedonobacteria* strains.

TABLE S4 *cox* gene **(A)** and KEGG pathway **(B)** analysis of the *Ktedonobacteria* strains.

TABLE S5 Pan-genome analysis of the *Ktedonobacteria* genomes **(A)**, *D. aurantiacus* S27^T^ and *Ktedonobacterales* bacterium Uno16 contig1 **(B)**, and *D. aurantiacus* S27^T^ and *Ktedonobacterales* bacterium Uno16 contig2 **(C)**.

TABLE S6 List of the putative BGCs identified by ClusterFinder **(A)** and antiSMASH **(B)** in nine *Ktedonobacteria* strains and their similarities to known clusters.

TABLE S7 Domain composition and organization of the putative NRPS clusters **(A)**, T1PKS clusters **(B)**, T2PKS clusters **(C)**, and NRPS-TIPKS clusters **(D)** identified in nine *Ktedonobacteria* strains, and AMP-binding domain amino acid substrate specificities in the NRPS-TIPKS clusters **(E)**.

## References

[B1] Adamek M. Alanjary M. Sales-Ortells H. Goodfellow M. Bull A. T. Winkler A. (2018). Comparative genomics reveals phylogenetic distribution patterns of secondary metabolites in *Amycolatopsis* species. *BMC Genomics* 19:426. 10.1186/s12864-018-4809-4 29859036 PMC5984834

[B2] Arce-Rodríguez A. Puente-Sánchez F. Avendaño R. Martínez-Cruz M. de Moor J. M. Pieper D. H. (2019). *Thermoplasmatales* and sulfur-oxidizing bacteria dominate the microbial community at the surface water of a CO2-rich hydrothermal spring located in Tenorio Volcano National Park, Costa Rica. *Extremophiles* 23 177–187. 10.1007/s00792-018-01072-6 30600357

[B3] Bérdy J. (2012). Thoughts and facts about antibiotics: where we are now and where we are heading. *J. Antibiot. (Tokyo)* 65 385–395. 10.1038/ja.2012.27 22511224

[B4] Bitok J. K. Lemetre C. Ternei M. A. Brady S. F. (2017). Identification of biosynthetic gene clusters from metagenomic libraries using PPTase complementation in a *Streptomyces* host. *FEMS Microbiol. Lett.* 364:fnx155. 10.1093/femsle/fnx155 28817927 PMC5827622

[B5] Björnsson L. Hugenholtz P. Tyson G. W. Blackall L. L. (2002). Filamentous *Chloroflexi* (green non-sulfur bacteria) are abundant in wastewater treatment processes with biological nutrient removal. *Microbiology* 148 2309–2318. 10.1099/00221287-148-8-2309 12177325

[B6] Blin K. Wolf T. Chevrette M. G. Lu X. Schwalen C. J. Kautsar S. A. (2017). antiSMASH 4.0 — Improvements in chemistry prediction and gene cluster boundary identification. *Nucleic Acids Res.* 45 W36–W41. 10.1093/nar/gkx319 28460038 PMC5570095

[B7] Bloudoff K. Fage C. D. Marahiel M. A. Schmeing T. M. (2017). Structural and mutational analysis of the nonribosomal peptide synthetase heterocyclization domain provides insight into catalysis. *Proc. Natl. Acad. Sci. U.S.A.* 114 95–100. 10.1073/pnas.1614191114 27994138 PMC5224394

[B8] Buchanan B. B. Arnon D. I. (1990). A reverse KREBS cycle in photosynthesis: consensus at last. *Photosynth. Res.* 24 47–53. 10.1007/BF00032643 11540925

[B9] Cavaletti L. Monciardini P. Bamonte R. Schumann P. Rohde M. Sosio M. (2006). New lineage of filamentous, spore-forming, gram-positive bacteria from soil. *Appl. Environ. Microbiol.* 72 4360–4369. 10.1128/AEM.00132-06 16751552 PMC1489649

[B10] Challis G. L. Naismith J. H. (2004). Structural aspects of non-ribosomal peptide biosynthesis. *Curr. Opin. Struct. Biol.* 14 748–756. 10.1016/j.sbi.2004.10.005 15582399 PMC3326538

[B11] Chang Y. J. Land M. Hauser L. Chertkov O. Del Rio T. G. Nolan M. (2011). Non-contiguous finished genome sequence and contextual data of the filamentous soil bacterium *Ktedonobacter racemifer* type strain (SOSP1-21). *Stand. Genomic Sci.* 5 97–111. 10.4056/sigs.2114901 22180814 PMC3236041

[B12] Chater K. F. Chandra G. (2006). The evolution of development in *Streptomyces* analysed by genome comparisons. *FEMS Microbiol. Rev.* 30 651–672. 10.1111/j.1574-6976.2006.00033.x 16911038

[B13] Chaudhari N. M. Gupta V. K. Dutta C. (2016). BPGA- an ultra-fast pan-genome analysis pipeline. *Sci. Rep.* 6:24373. 10.1038/srep24373 27071527 PMC4829868

[B14] Chen L. Y. Lai Y. M. Yang Y. L. Zhao X. Q. (2016). Genome mining reveals the biosynthetic potential of the marinederived strain *Streptomyces marokkonensis* M10. *Synth. Syst. Biotechnol.* 1 56–65. 10.1016/j.synbio.2016.02.005 29062928 PMC5640592

[B15] Chen W. H. Li K. Guntaka N. S. Bruner S. D. (2016). Interdomain and intermodule organization in epimerization domain containing nonribosomal peptide synthetases. *ACS Chem. Biol.* 11 2293–2303. 10.1021/acschembio.6b00332 27294598

[B16] Chevrette M. G. Aicheler F. Kohlbacher O. Currie C. R. Medema M. H. (2017). SANDPUMA: ensemble predictions of nonribosomal peptide chemistry reveal biosynthetic diversity across *Actinobacteria*. *Bioinformatics* 33 3202–3210. 10.1093/bioinformatics/btx400 28633438 PMC5860034

[B17] Chu J. Vila-Farres X. Inoyama D. Ternei M. Cohen L. J. Gordon E. A. (2016). Discovery of MRSA active antibiotics using primary sequence from the human microbiome. *Nat. Chem. Biol.* 12 1004–1006. 10.1038/nchembio.2207 27748750 PMC5117632

[B18] Cimermancic P. Medema M. H. Claesen J. Kurita K. Wieland Brown L. C. (2014). Insights into secondary metabolism from a global analysis of prokaryotic biosynthetic gene clusters. *Cell* 158 412–421. 10.1016/j.cell.2014.06.034 25036635 PMC4123684

[B19] Crooks G. E. Hon G. Chandonia J. M. Brenner S. E. (2004). WebLogo: a sequence logo generator. *Genome Res.* 14 1188–1190. 10.1101/gr.849004 15173120 PMC419797

[B20] Darling A. C. Mau B. Blattner F. R. Perna N. T. (2004). Mauve: multiple alignment of conserved genomic sequence with rearrangements. *Genome Res.* 14 1394–1403. 10.1101/gr.2289704 15231754 PMC442156

[B21] Darling A. E. Jospin G. Lowe E. Matsen F. A. IV Bik H. M. Eisen J. A. (2014). PhyloSift: phylogenetic analysis of genomes and metagenomes. *PeerJ.* 2:e243. 10.7717/peerj.243 24482762 PMC3897386

[B22] Demain A. L. Sanchez S. (2009). Microbial drug discovery: 80 years of progress. *J. Antibiot. (Tokyo)* 62 5–16. 10.1038/ja.2008.16 19132062 PMC7094699

[B23] de Miera L. E. S. Arroyo P. de Luis Calabuig E. Ansola G. (2014a). Effects of varying CO2 flows on bacterial communities in mesocosms created from two soils. *Int. J. Greenhouse Gas Control* 46 205–214. 10.1016/j.ijggc.2016.01.013

[B24] de Miera L. E. S. Arroyo P. de Luis Calabuig E. Falagán J. Ansola G. (2014b). High-throughput sequencing of 16S RNA genes of soil bacterial communities from a naturally occurring CO_2_ gas vent. *Int. J. Greenhouse Gas Conrol* 29 176–184. 10.1016/j.ijggc.2014.08.014

[B25] Domalaon R. Idowu T. Zhanel G. G. Schweizer F. (2018). Antibiotic hybrids: the next generation of agents and adjuvants against gram-negative pathogens? *Clin. Microbiol. Rev.* 31:e00077-17. 10.1128/CMR.00077-17 29540434 PMC5967690

[B26] Donadio S. Monciardini P. Sosio M. (2007). Polyketide synthases and nonribosomal peptide synthetases: the emerging view from bacterial genomics. *Nat. Prod. Rep.* 24 1073–1109. 10.1039/b514050c 17898898

[B27] Doroghazi J. R. Metcalf W. W. (2013). Comparative genomics of actinomycetes with a focus on natural product biosynthetic genes. *BMC Genomics* 14:611. 10.1186/1471-2164-14-611 24020438 PMC3848822

[B28] Dutta S. Whicher J. R. Hansen D. A. Hale W. A. Chemler J. A. Congdon G. R. (2014). Structure of a modular polyketide synthase. *Nature* 510 512–517. 10.1038/nature13423 24965652 PMC4278352

[B29] Dziewit L. Pyzik A. Szuplewska M. Matlakowska R. Mielnicki S. Wibberg D. (2015). Diversity and role of plasmids in adaptation of bacteria inhabiting the Lubin copper mine in Poland, an environment rich in heavy metals. *Front. Microbiol.* 3:152. 10.3389/fmicb.2015.00152 26074880 PMC4447125

[B30] Errington J. (2003). Regulation of endospore formation in *Bacillus subtilis*. *Nat. Rev. Microbiol.* 1 117–126. 10.1038/nrmicro750 15035041

[B31] Finn R. D. Coggill P. Eberhardt R. Y. Eddy S. R. Mistry J. Mitchell A. L. (2016). The Pfam protein families database: towards a more sustainable future. *Nucleic Acids Res.* 44 D279–D285. 10.1093/nar/gkv1344 26673716 PMC4702930

[B32] Flärdh K. Buttner M. J. (2009). *Streptomyces morphogenetics*: dissecting differentiation in a filamentous bacterium. *Nat. Rev. Microbiol.* 7 36–49. 10.1038/nrmicro1968 19079351

[B33] Galperin M. Y. (2001). Conserved ‘hypothetical’ proteins: new hints and new puzzles. *Comp. Funct. Genomics* 2 14–18. 10.1002/cfg.66 18628897 PMC2447192

[B34] Genilloud O. (2017). Actinomycetes: still a source of novel antibiotics. *Nat. Prod. Rep.* 34 1203–1232. 10.1039/c7np00026j 28820533

[B35] Grant J. R. Stothard P. (2008). The CGView server: a comparative genomics tool for circular genomes. *Nucleic Acids Res.* 36 W181–W184. 10.1093/nar/gkn179 18411202 PMC2447734

[B36] Gupta R. S. Chander P. George S. (2013). Phylogenetic framework and molecular signatures for the class *Chloroflexi* and its different clades; proposal for division of the class Chloroflexia class. nov. into the suborder *Chloroflexineae* subord. nov., consisting of the emended family *Oscillochloridaceae* and the family *Chloroflexaceae* fam. nov., and the suborder *Roseiflexineae* subord. nov., containing the family *Roseiflexaceae* fam. nov. *Antonie Van Leeuwenhoek* 103 99–119. 10.1007/s10482-012-9790-3 22903492

[B37] Han K. Li Z. F. Peng R. Zhu L. P. Zhou T. Wang L. G. (2013). Extraordinary expansion of a *Sorangium cellulosum* genome from an alkaline milieu. *Sci. Rep.* 3:2101. 10.1038/srep02101 23812535 PMC3696898

[B38] Hayashi T. Kitamura Y. Funa N. Ohnishi Y. Horinouchi S. (2011). Fatty acyl-AMP ligase involvement in the production of alkylresorcylic acid by a *Myxococcus xanthus* type III polyketide synthase. *Chembiochem* 12 2166–2176. 10.1002/cbic.201100344 21815236

[B39] Hisanaga Y. Ago H. Nakagawa N. Hamada K. Ida K. Yamamoto M. (2004). Structural basis of the substrate-specific two-step catalysis of long chain fatty acyl-CoA synthetase dimer. *J. Biol. Chem.* 279 31717–31726. 10.1074/jbc.M400100200 15145952

[B40] Hug L. A. Baker B. J. Anantharaman K. Brown C. T. Probst A. J. Castelle C. J. (2016). A new view of the tree of life. *Nat. Microbiol.* 1:16048. 10.1038/nmicrobiol.2016.48 27572647

[B41] Ibal J. C. Pham H. Q. Park C. E. Shin J. H. (2019). Information about variations in multiple copies of bacterial 16S rRNA genes may aid in species identification. *PLoS One* 14:e0212090. 10.1371/journal.pone.0212090 30768621 PMC6377111

[B42] Islam Z. F. Cordero P. F. R. Feng J. Chen Y.-J. Bay S. K. Jirapanjawat T. (2019). Two *Chloroflexi* classes independently evolved the ability to persist on atmospheric hydrogen and carbon monoxide. *ISME J.* 10.1038/s41396-019-0393-0 30872805 PMC6776052

[B43] Jenke-Kodama H. Dittmann E. (2009). Evolution of metabolic diversity: insights from microbial polyketide synthases. *Phytochemistry* 70 1858–1866. 10.1016/j.phytochem.2009.05.021 19619887

[B44] Jenke-Kodama H. Sandmann A. Müller R. Dittmann E. (2005). Evolutionary implications of bacterial polyketide synthases. *Mol. Biol. Evol.* 22 2027–2039. 10.1093/molbev/msi193 15958783

[B45] Jensen P. R. (2016). Natural products and the gene cluster revolution. *Trends Microbiol.* 24 968–977. 10.1016/j.tim.2016.07.006 27491886 PMC5123934

[B46] Jiang X. Ellabaan M. M. Charusanti P. Munck C. Blin K. Tong Y. (2017). Dissemination of antibiotic resistance genes from antibiotic producers to pathogens. *Nat. Commun.* 8:15784. 10.1038/ncomms15784 28589945 PMC5467266

[B47] Jiang Z. Li P. Jiang D. Dai X. Zhang R. Wang Y. (2016). Microbial community structure and arsenic biogeochemistry in an acid vapor-formed spring in Tengchong Geothermal Area, China. *PLoS One* 11:e0146331. 10.1371/journal.pone.0146331 26761709 PMC4711897

[B48] Johnston C. W. Skinnider M. A. Wyatt M. A. Li X. Ranieri M. R. Yang L. (2015). An automated genomes-to-natural products platform (GNP) for the discovery of modular natural products. *Nat. Commun.* 6:8421. 10.1038/ncomms9421 26412281 PMC4598715

[B49] Kaster A. K. Mayer-Blackwell K. Pasarelli B. Spormann A. M. (2014). Single cell genomic study of *Dehalococcoidetes* species from deep-sea sediments of the Peruvian Margin. *ISME J.* 8 1831–1842. 10.1038/ismej.2014.24 24599070 PMC4139717

[B50] Kelly S. L. Kelly D. E. (2013). Microbial cytochromes P450: biodiversity and biotechnology. Where do cytochromes P450 come from, what do they do and what can they do for us? *Philos. Trans. R. Soc. Lond. B Biol. Sci.* 6:358. 10.1098/rstb.2012.0476 23297358 PMC3538425

[B51] King C. E. King G. M. (2014). Description of *Thermogemmatispora carboxidivorans* sp. nov., a carbon-monoxide-oxidizing member of the class *Ktedonobacteria* isolated from a geothermally heated biofilm, and analysis of carbon monoxide oxidation by members of the class *Ktedonobacteria*. *Int. J. Syst. Evol. Microbiol.* 64 1244–1251. 10.1099/ijs.0.059675-0 24425739

[B52] Komaki H. Hosoyama A. Yabe S. Yokota A. Uchino Y. Takano H. (2016). Draft genome sequence of *Thermogemmatispora onikobensis* NBRC 111776T, an aerial mycelium- and spore-forming thermophilic bacterium belonging to the class *Ktedonobacteria*. *Genome Announc.* 4:e1156-16. 10.1128/genomeA.01156-16 27738048 PMC5064121

[B53] Kuroda M. Ohta T. Uchiyama I. Baba T. Yuzawa H. Kobayashi I. (2001). Whole genome sequencing of meticillin-resistant *Staphylococcus aureus*. *Lancet* 357 1225–1240. 10.1016/S0140-6736(00)04403-2 11418146

[B54] Landry Z. Swan B. K. Herndl G. J. Stepanauskas R. Giovannoni S. J. (2017). SAR202 genomes from the dark ocean predict pathways for the oxidation of recalcitrant dissolved organic matter. *MBio* 8 e413–e417. 10.1128/mBio.00413-17 28420738 PMC5395668

[B55] Lawrence J. (1999). Selfish operons: the evolutionary impact of gene clustering in prokaryotes and eukaryotes. *Curr. Opin. Genet. Dev.* 9 642–648. 10.1016/S0959-437X(99)00025-8 10607610

[B56] Lázár V. Martins A. Spohn R. Daruka L. Grézal G. Fekete G. (2018). Antibiotic-resistant bacteria show widespread collateral sensitivity to antimicrobial peptides. *Nat. Microbiol.* 3 718–731. 10.1038/s41564-018-0164-0 29795541 PMC6544545

[B57] Letunic I. Bork P. (2016). Interactive tree of life (iTOL) v3: an online tool for the display and annotation of phylogenetic and other trees. *Nucleic Acids Res.* 44 W242–W245. 10.1093/nar/gkw290 27095192 PMC4987883

[B58] Letzel A. C. Pidot S. J. Hertweck C. (2014). Genome mining for ribosomally synthesized and post-translationally modified peptides (RiPPs) in anaerobic bacteria. *BMC Genomics* 15:983. 10.1186/1471-2164-15-983 25407095 PMC4289311

[B59] Li B. Walsh C. T. (2010). Identification of the gene cluster for the dithiolopyrrolone antibiotic holomycin in *Streptomyces clavuligerus*. *Proc. Natl. Acad. Sci. U.S.A.* 107 19731–19735. 10.1073/pnas.1014140107 21041678 PMC2993409

[B60] Liu F. Garneau S. Walsh C. T. (2004). Hybrid nonribosomal peptide-polyketide interfaces in epothilone biosynthesis: minimal requirements at N and C termini of EpoB for elongation. *Chem. Biol.* 11 1533–1542. 10.1016/j.chembiol.2004.08.017 15556004

[B61] Ludwig W. Strunk O. Westram R. Richter L. Meier H. Yadhukumar (2004). ARB: a software environment for sequence data. *Nucleic Acids Res.* 32 1363–1371. 10.1093/nar/gkh293 14985472 PMC390282

[B62] Manivasagan P. Venkatesan J. Sivakumar K. Kim S. K. (2014). Pharmaceutically active secondary metabolites of marine actinobacteria. *Microbiol. Res.* 169 262–278. 10.1016/j.micres.2013.07.014 23958059

[B63] Masschelein J. Jenner M. Challis G. L. (2017). Antibiotics from gram-negative bacteria: a comprehensive overview and selected biosynthetic highlights. *Nat. Prod. Rep.* 34 712–783. 10.1039/c7np00010c 28650032

[B64] Miller D. A. Luo L. Hillson N. Keating T. A. Walsh C. T. (2002). Yersiniabactin synthetase: a four-protein assembly line producing the nonribosomal peptide/polyketide hybrid siderophore of *Yersinia pestis*. *Chem. Biol.* 9 333–344. 11927258 10.1016/s1074-5521(02)00115-1

[B65] Miller S. I. (2016). Antibiotic resistance and regulation of the gram-negative bacterial outer membrane barrier by host innate immune molecules. *MBio* 7 e1541–e1516. 10.1128/mBio.01541-16 27677793 PMC5040116

[B66] Moe W. M. Yan J. Nobre M. F. da Costa M. S. Rainey F. A. (2009). *Dehalogenimonas lykanthroporepellens* gen. nov., sp. nov., a reductively dehalogenating bacterium isolated from chlorinated solvent-contaminated groundwater. *Int. J. Syst. Evol. Microbiol.* 59 2692–2697. 10.1099/ijs.0.011502-0 19625421

[B67] Moriya Y. Itoh M. Okuda S. Yoshizawa A. Kanehisa M. (2007). KAAS: an automatic genome annotation and pathway reconstruction server. *Nucleic Acids Res.* 35 W182–W185. 10.1093/nar/gkm321 17526522 PMC1933193

[B68] Naughton L. M. Romano S. O’Gara F. Dobson A. D. W. (2017). Identification of secondary metabolite gene clusters in the *Pseudovibrio* genus reveals encouraging biosynthetic potential toward the production of novel bioactive compounds. *Front. Microbiol.* 8:1494. 10.3389/fmicb.2017.01494 28868049 PMC5563371

[B69] Northup D. E. Melim L. A. Spilde M. N. Hathaway J. J. Garcia M. G. Moya M. (2011). Lava cave microbial communities within mats and secondary mineral deposits: implications for life detection on other planets. *Astrobiology* 11 601–618. 10.1089/ast.2010.0562 21879833 PMC3176350

[B70] Okada Y. (1937). Occurrence of masses of gelatinous microbes in the soil. *Soil Sci.* 43 367–374. 10.1097/00010694-193705000-00005

[B71] Omura S. Ikeda H. Ishikawa J. Hanamoto A. Takahashi C. Shinose M. (2001). Genome sequence of an industrial microorganism *Streptomyces avermitilis*: deducing the ability of producing secondary metabolites. *Proc. Natl. Acad. Sci. U.S.A.* 98 12215–12220. 10.1073/pnas.211433198 11572948 PMC59794

[B72] Ortega M. A. Hao Y. Zhang Q. Walker M. C. van der Donk W. A. Nair S. K. (2015). Structure and mechanism of the tRNA-dependent lantibiotic dehydratase NisB. *Nature* 517 509–512. 10.1038/nature13888 25363770 PMC4430201

[B73] Ortega M. A. van der Donk W. A. (2016). New insights into the biosynthetic logic of ribosomally synthesized and post-translationally modified peptide natural products. *Cell Chem. Biol.* 23 31–44. 10.1016/j.chembiol.2015.11.012 26933734 PMC4779184

[B74] Pal C. Bengtsson-Palme J. Kristiansson E. Larsson D. G. (2016). The structure and diversity of human, animal and environmental resistomes. *Microbiome* 4:54. 10.1186/s40168-016-0199-5 27717408 PMC5055678

[B75] Park J. S. Kagaya N. Hashimoto J. Izumikawa M. Yabe S. Shin-ya K. (2014). Identification and biosynthesis of new acyloins from the thermophilic bacterium *Thermosporothrix hazakensis* SK20-1(T). *Chembiochem* 15 527–532. 10.1002/cbic.201300690 24474719

[B76] Park J. S. Yabe S. Shin-ya K. Nishiyama M. Kuzuyama T. (2015). New 2-(1’H-indole-3’-carbonyl)-thiazoles derived from the thermophilic bacterium *Thermosporothrix hazakensis* SK20-1(T). *J. Antibiot. (Tokyo)* 68 60–62. 10.1038/ja.2014.93 25052483

[B77] Parks D. H. Imelfort M. Skennerton C. T. Hugenholtz P. Tyson G. W. (2015). CheckM: assessing the quality of microbial genomes recovered from isolates, single cells, and metagenomes. *Genome Res.* 25 1043–1055. 10.1101/gr.186072.114 25977477 PMC4484387

[B78] Price M. N. Dehal P. S. Arkin A. P. (2010). FastTree 2–approximately maximum-likelihood trees for large alignments. *PLoS One* 5:e9490. 10.1371/.journal.pone.0009490 20224823 PMC2835736

[B79] Rausch C. Hoof I. Weber T. Wohlleben W. Huson D. H. (2007). Phylogenetic analysis of condensation domains in NRPS sheds light on their functional evolution. *BMC Evol. Biol.* 7:78. 10.1186/1471-2148-7-78 17506888 PMC1894796

[B80] Romaniuk K. Golec P. Dziewit L. (2018). Insight into the diversity and possible role of plasmids in the adaptation of psychrotolerant and metalotolerant *Arthrobacter* spp. to extreme Antarctic environments. *Front. Microbiol.* 9:3144. 10.3389/fmicb.2018.03144 30619210 PMC6305408

[B81] Röttig M. Medema M. H. Blin K. Weber T. Rausch C. Kohlbacher O. (2011). NRPSpredictor2–a web server for predicting NRPS adenylation domain specificity. *Nucleic Acids Res.* 39 W362–W367. 10.1093/nar/gkr323 21558170 PMC3125756

[B82] Rutledge P. J. Challis G. L. (2015). Discovery of microbial natural products by activation of silent biosynthetic gene clusters. *Nat. Rev. Microbiol.* 13 509–523. 10.1038/nrmicro3496 26119570

[B83] Sabath N. Ferrada E. Barve A. Wagner A. (2013). Growth temperature and genome size in bacteria are negatively correlated, suggesting genomic streamlining during thermal adaptation. *Genome Biol. Evol.* 5 966–977. 10.1093/gbe/evt050 23563968 PMC3673621

[B84] Sekiguchi Y. Ohashi A. Parks D. H. Yamauchi T. Tyson G. W. Hugenholtz P. (2015). First genomic insights into members of a candidate bacterial phylum responsible for wastewater bulking. *PeerJ* 3:e740. 10.7717/peerj.740 25650158 PMC4312070

[B85] Shen B. (2003). Polyketide biosynthesis beyond the type I, II and III polyketide synthase paradigms. *Curr. Opin. Chem. Biol.* 7 285–295. 10.1016/s1367-5931(03)00020-6 12714063

[B86] Shirling E. B. Gottlieb D. (1966). Methods for characterization of *Streptomyces* species. *Int. J. Syst. Bacteriol.* 16 313–340. 10.1099/00207713-16-3-313

[B87] Shou Q. Feng L. Long Y. Han J. Nunnery J. K. Powell D. H. (2016). A hybrid polyketide-nonribosomal peptide in nematodes that promotes larval survival. *Nat. Chem. Biol.* 12 770–772. 10.1038/nchembio.2144 27501395 PMC5030153

[B88] Soo R. M. Skennerton C. T. Sekiguchi Y. Imelfort M. Paech S. J. Dennis P. G. (2014). An expanded genomic representation of the phylum *Cyanobacteria*. *Genome Biol. Evol.* 6 1031–1045. 10.1093/gbe/evu073 24709563 PMC4040986

[B89] Soupene E. Kuypers F. A. (2008). Mammalian long-chain acyl-CoA synthetases. *Exp. Biol. Med. (Maywood)* 233 507–521. 10.3181/0710-MR-287 18375835 PMC3377585

[B90] Stachelhaus T. Mootz H. D. Marahiel M. A. (1999). The specificity-conferring code of adenylation domains in nonribosomal peptide synthetases. *Chem. Biol.* 6 493–505. 10.1016/S1074-5521(99)80082-9 10421756

[B91] Stamatakis A. (2006). RAxML-VI-HPC: maximum likelihood-based phylogenetic analyses with thousands of taxa and mixed models. *Bioinformatics* 22 2688–2690. 10.1093/bioinformatics/btl446 16928733

[B92] Stres B. Sul W. J. Murovec B. Tiedje J. M. (2013). Recently deglaciated high-altitude soils of the Himalaya: diverse environments, heterogenous bacterial communities and long-range dust inputs from the upper troposphere. *PLoS One* 8:e76440. 10.1371/journal.pone.0076440 24086740 PMC3784432

[B93] Sun L. Toyonaga M. Ohashi A. Tourlousse D. M. Matsuura N. Meng X. Y. (2016). *Lentimicrobium saccharophilum* gen. nov., sp. nov., a strictly anaerobic bacterium representing a new family in the phylum *Bacteroidetes*, and proposal of *Lentimicrobiaceae fam*. nov. *Int. J. Syst. Evol. Microbiol.* 66 2635–2642. 10.1099/ijsem.0.001103 27098854

[B94] Swofford D. L. (2003). *PAUP^∗^: Phylogenetic Analysis Using Parsimony, Version 4.0b10*. Available at: https://paup.phylosolutions.com/ (accessed September 2018).

[B95] Tanizawa Y. Fujisawa T. Nakamura Y. (2018). DFAST: a flexible prokaryotic genome annotation pipeline for faster genome publication. *Bioinformatics* 34 1037–1039. 10.1093/bioinformatics/btx713 29106469 PMC5860143

[B96] Tebo B. M. Davis R. E. Anitori R. P. Connell L. B. Schiffman P. Staudigel H. (2015). Microbial communities in dark oligotrophic volcanic ice cave ecosystems of Mt. Erebus, Antarctica. *Front. Microbiol.* 6:179 10.3389/fmicb.2015.00179 PMC4356161 25814983

[B97] Tettelin H. Masignani V. Cieslewicz M. J. Donati C. Medini D. Ward N. L. (2005). Genome analysis of multiple pathogenic isolates of *Streptococcus agalactiae*: implications for the microbial “pan-genome”. *Proc. Natl. Acad. Sci. U.S.A.* 102 13950–13955. 10.1073/pnas.0506758102 16172379 PMC1216834

[B98] Thomas C. M. Summers D. (2008). “Bacterial plasmids,” in *Encyclopedia of Life Sciences (ELS)*, Chichester: John Wiley & Sons, Ltd 10.1002/9780470015902.a0000468.pub2

[B99] Tooming-Klunderud A. Sogge H. Rounge T. B. Nederbragt A. J. Lagesen K. Glöckner G. (2013). From green to red: horizontal gene transfer of the phycoerythrin gene cluster between *Planktothrix* strains. *Appl. Environ. Microbiol.* 79 6803–6812. 10.1128/AE01455-13 23995927 PMC3811498

[B100] van Heel A. J. de Jong A. Montalbán-López M. Kok J. Kuipers O. P. (2013). BAGEL3: automated identification of genes encoding bacteriocins and (non-)bactericidal posttranslationally modified peptides. *Nucleic Acids Res.* 41 W448–W453. 10.1093/nar/gkt391 23677608 PMC3692055

[B101] Vila-Farres X. Chu J. Inoyama D. Ternei M. A. Lemetre C. Cohen L. J. (2017). Antimicrobials inspired by nonribosomal peptide synthetase gene clusters. *J. Am. Chem. Soc.* 139 1404–1407. 10.1021/jacs.6b11861 28055186 PMC7163397

[B102] Wang C. M. Zheng Y. Sakai Y. Toyoda A. Minakuchi Y. Abe K. (2019). *Tengunoibacter tsumagoiensis* gen. nov., sp. nov., *Dictyobacter kobayashii* sp. nov., *Dictyobacter alpinus* sp. nov., and description of *Dictyobacteraceae* fam. nov. within the order *Ktedonobacterales* isolated from Tengu-no-mugimeshi, a soil-like granular mass of microorganisms, and emended descriptions of the genera *Ktedonobacter* and *Dictyobacter*. *Int. J. Syst. Evol. Microbiol*. 69 (in press). 10.1099/ijsem.0.003396 30990396

[B103] Willey J. M. van der Donk W. A. (2007). Lantibiotics: peptides of diverse structure and function. *Annu. Rev. Microbiol.* 61 477–501. 10.1146/annurev.micro.61.080706.093501 17506681

[B104] Wu D. Raymond J. Wu M. Chatterji S. Ren Q. Graham J. E. (2009). Complete genome sequence of the aerobic CO-oxidizing thermophile *Thermomicrobium roseum*. *PLoS One* 4:e4207. 10.1371/journal.pone.0004207 19148287 PMC2615216

[B105] Xu B. Aitken E. J. Baker B. P. Turner C. A. Harvey J. E. Stott M. B. (2018). Genome mining, isolation, chemical synthesis and biological evaluation of a novel lanthipeptide, tikitericin, from the extremophilic microorganism *Thermogemmatispora* strain T81. *Chem. Sci.* 9 7311–7317. 10.1039/c8sc02170h 30294420 PMC6167946

[B106] Yabe S. Aiba Y. Sakai Y. Hazaka M. Yokota A. (2010). *Thermosporothrix hazakensis* gen. nov., sp. nov., isolated from compost, description of *Thermosporotrichaceae* fam. nov. within the class *Ktedonobacteria* Cavaletti et al. 2007 and emended description of the class *Ktedonobacteria*. *Int. J. Syst. Evol. Microbiol.* 60 1794–1801. 10.1099/ijs.0.018069-0 19767365

[B107] Yabe S. Aiba Y. Sakai Y. Hazaka M. Yokota A. (2011). *Thermogemmatispora onikobensis* gen. nov., sp. nov. and *Thermogemmatispora foliorum* sp. nov., isolated from fallen leaves on geothermal soils, and description of *Thermogemmatisporaceae* fam. nov. and *Thermogemmatisporales* ord. nov. within the class *Ktedonobacteria*. *Int. J. Syst. Evol. Microbiol.* 61 903–910. 10.1099/ijs.0.024877-0 20495028

[B108] Yabe S. Sakai Y. Abe K. Yokota A. (2017a). Diversity of *Ktedonobacteria* with actinomycetes-like morphology in terrestrial environments. *Microbes Environ.* 31 61–70. 10.1264/jsme2.ME16144 28321007 PMC5371077

[B109] Yabe S. Sakai Y. Abe K. Yokota A. Také A. Matsumoto A. (2017b). *Dictyobacter aurantiacus* gen. nov., sp. nov., a member of the family *Ktedonobacteraceae*, isolated from soil, and emended description of the genus *Thermosporothrix*. *Int. J. Syst. Evol. Microbiol.* 67 2615–2621. 10.1099/ijsem.0.001985 28758628

[B110] Yabe S. Wang C. M. Zheng Y. Sakai Y. Abe K. Yokota A. (2019). Formation of sporangiospores in *Dictyobacter aurantiacus* (class *Ktedonobacteria* in phylum *Chloroflexi*). *J. Gen. Appl. Microbiol.* 65 (in press). 10.2323/jgam.2019.01.001 31118349

[B111] Yarza P. Ludwig W. Euzéby J. Amann R. Schleifer K. H. Glöckner F. O. (2010). Update of the all-species living tree project based on 16S and 23S rRNA sequence analyses. *Syst. Appl. Microbiol.* 33 291–299. 10.1016/j.syapm.2010.08.001 20817437

[B112] Zhang Q. Yu Y. Vélasquez J. E. van der Donk W. A. (2012). Evolution of lanthipeptide synthetases. *Proc. Natl. Acad. Sci. U.S.A.* 109 18361–18366. 10.1073/pnas.1210393109 23071302 PMC3494888

[B113] Ziemert N. Lechner A. Wietz M. Millán-Aguiñaga N. Chavarria K. L. Jensen P. R. (2014). Diversity and evolution of secondary metabolism in the marine actinomycete genus *Salinispora*. *Proc. Natl. Acad. Sci. U.S.A.* 111 E1130–E1139. 10.1073/pnas1324161111 24616526 PMC3970525

[B114] Ziemert N. Podell S. Penn K. Badger J. H. Allen E. Jensen P. R. (2012). The natural product domain seeker NaPDoS: a phylogeny based bioinformatic tool to classify secondary metabolite gene diversity. *PLoS One* 7:e34064. 10.1371/journal.pone.0034064 22479523 PMC3315503

