# Supplementary figures and images for: Genome Features and Secondary Metabolites Biosynthetic Potential of the Class Ktedonobacteria
Source: Front Microbiol. 2019 Apr 26;10:893. doi: 10.3389/fmicb.2019.00893 (PMC6497799; doi:10.3389/fmicb.2019.00893)

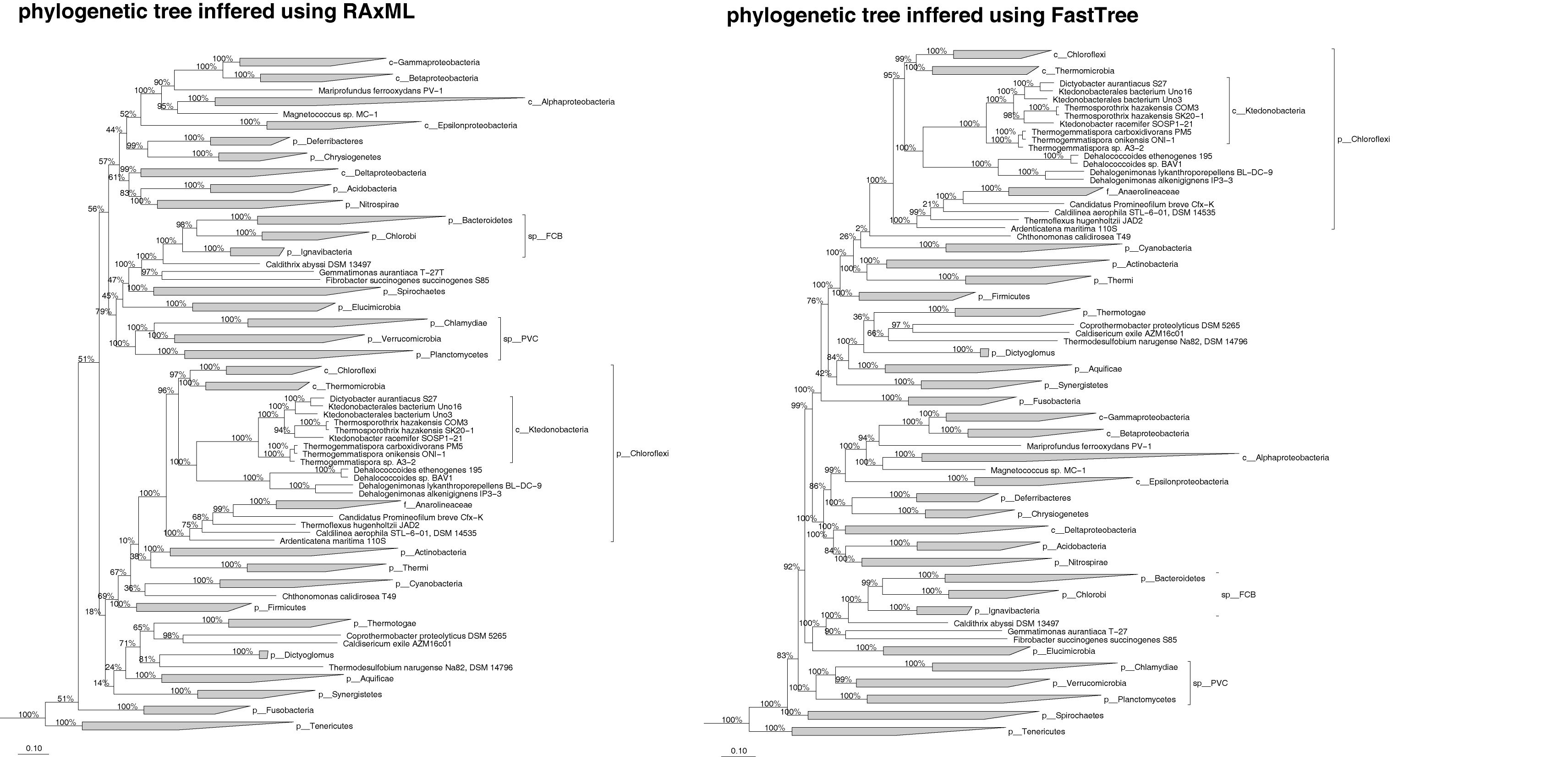

Supplement: FIGURE S1 — Maximum-likelihood phylogenetic inference of members of the class Ktedonobacteria among known bacterial phyla. The tree was constructed using RAxML or FastTree based on up to 38 marker genes (using taxon-outgroup configuration Config 3 described in Sekiguchi et al., 2015) and sequences were collapsed at the phylum level except for classes in the Proteobacteria. Ranks are indicated by prefix; p_ (phylum), c_ (class). Parameters used for phylogenetic inference using RAxML and FastTree are described in the “Materials and Methods” section. Numbers at the notes indicate bootstrap values (%) with 100 times determination. The scale bar represents 10% estimated sequence divergence. [file Image_1.TIF]

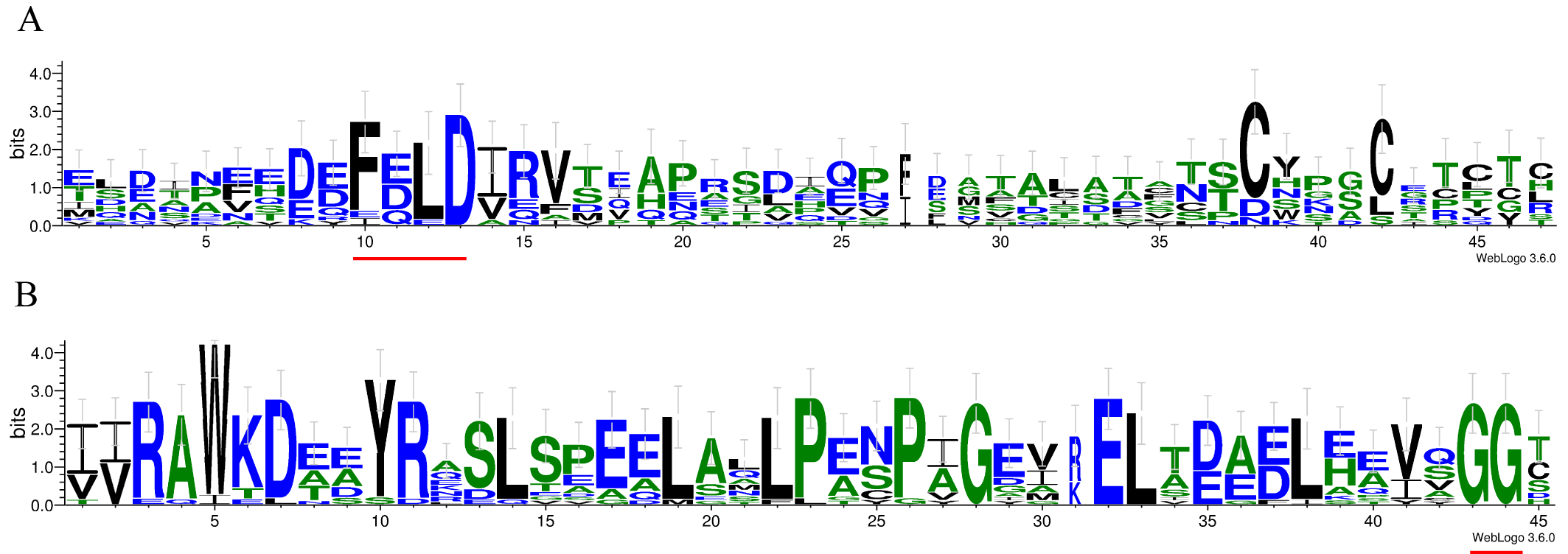

Supplement: FIGURE S2 — Sequence logos of precursor peptide conserved motifs identified in the nine Ktedonobacteria strains are underlined in red. (A) The “F(E/D)L” cleavage site for Class I lantipeptide gene clusters. (B) The “GG” cleavage site for Class II lantipeptide clusters. [file Image_2.TIF]

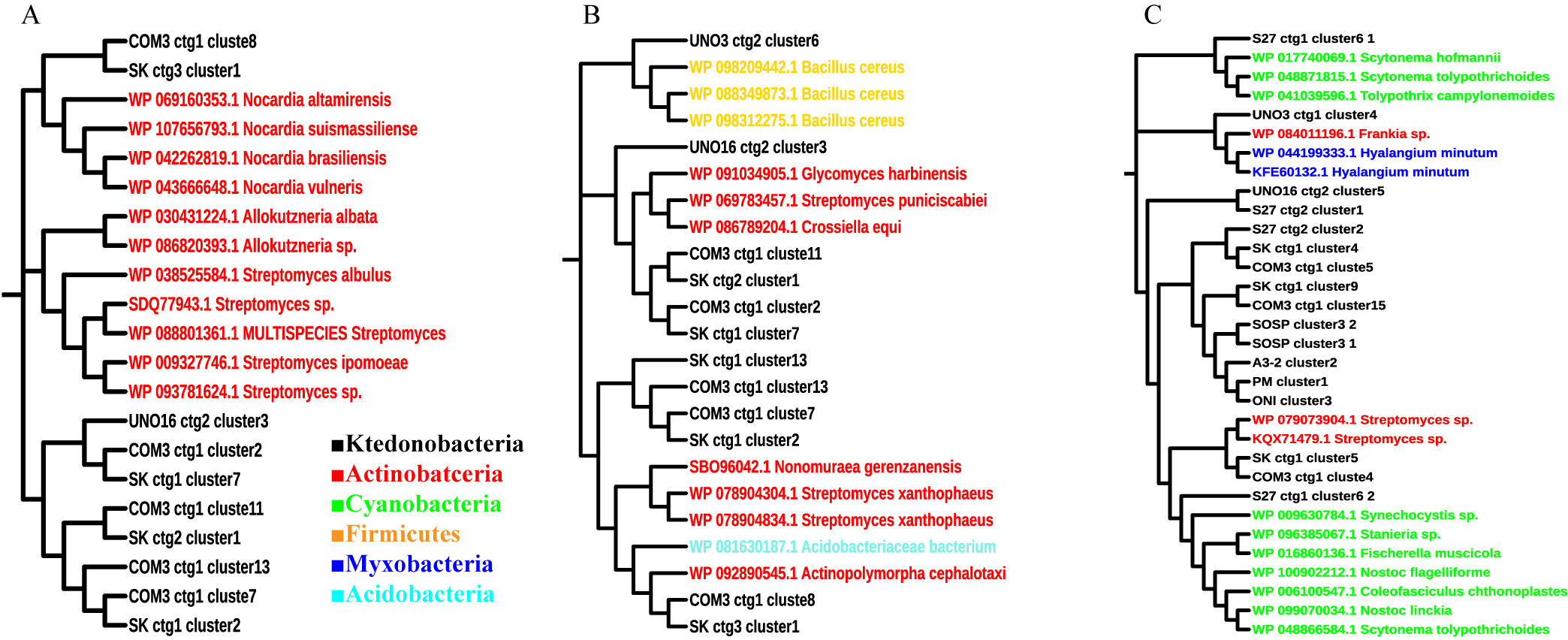

Supplement: FIGURE S3 — Phylogenetic analysis of the Ktedonobacteria modification genes in lantipeptide clusters. (A) Phylogenetic analysis of the Ktedonobacteria LanB genes. Amino acid sequences extracted from the Ktedonobacteria LanB and the three top hit reference sequences from Protein-Protein Blast were aligned by MEGA v. 7.0. Maximum-likelihood method was used to build the tree. The background colors represent evolutionary classification of the LanB modification genes. (B) Phylogenetic analysis of the Ktedonobacteria LanC genes. The tree was built as for (A). (C) Phylogenetic analysis of the Ktedonobacteria LanM genes. The tree was also built as for (A). [file Image_3.TIF]

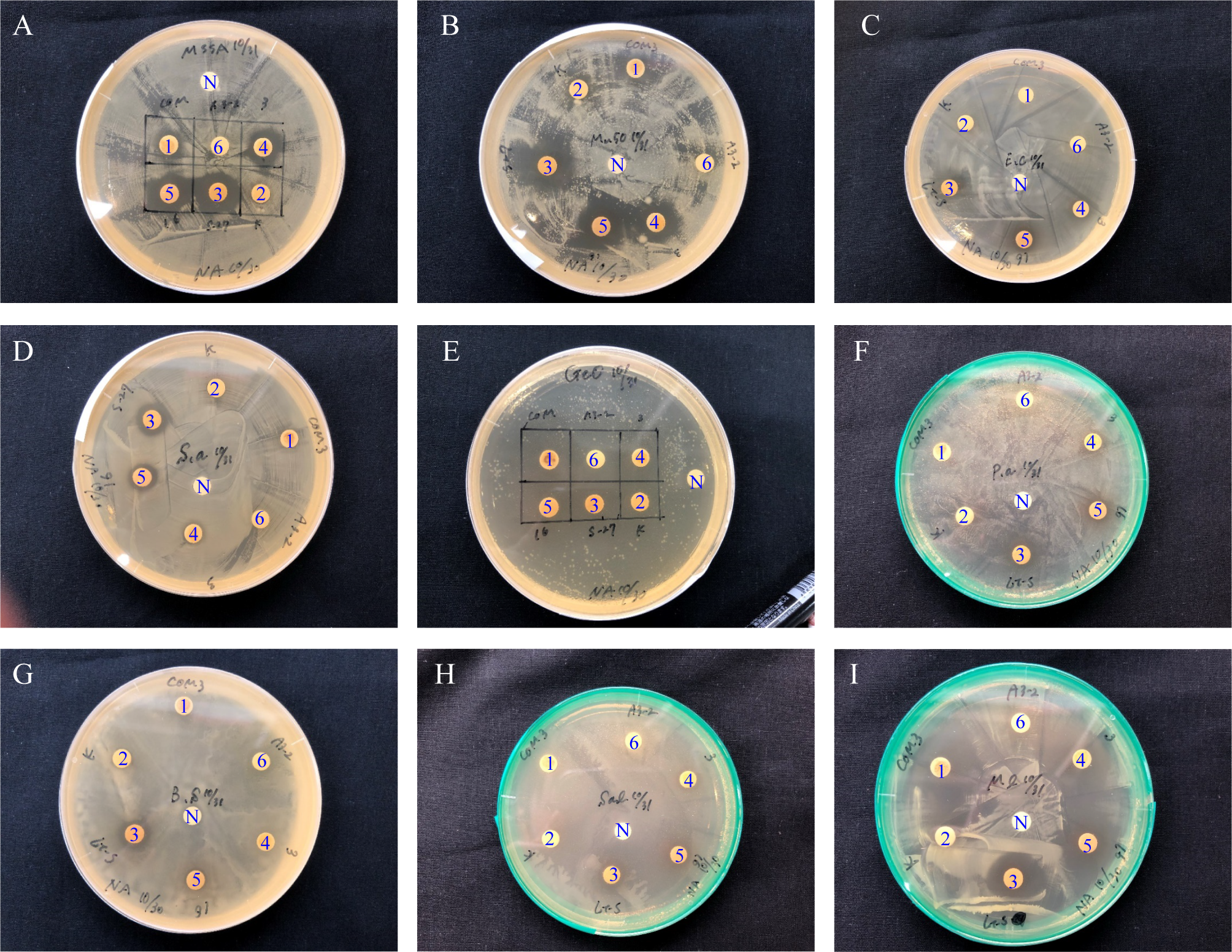

Supplement: FIGURE S4 — Antimicrobial screening of six representative Ktedonobacteria strains against bacterial and fungal strains. The antimicrobial activity are indicated by formation of clear zone of S. aureus NTCT8325 (MSSA) (A), S. aureus Mu50 (VISA) (B), Escherichia coli NBRC3972 (C), Bacillus subtilis NBRC3134 (D), Staphylococcus aureus NBRC13276 (E), Geobacillus stearothermophilus NBRC13737 (F), Pseudomonas aeruginosa NBRC13275 (G), Salmonella enterica NBRC100797 (H), Micrococcus luteus NBRC13867 (I) as inhibited by Thermosporothrix hazakensis COM3 (1), Ktedonobacter racemifer SOSP1-21T (2), Dictyobacter aurantiacus S27T (3), Ktedonobacterales bacterium Uno3 (4), Ktedonobacterales bacterium Uno16 (5), Thermogemmatispora sp. A3-2 (6), and negative control (N). [file Image_4.TIF]
